# Immune responses to inactivated COVID-19 vaccine were decreased in Chinese patients with chronic respiratory diseases

**DOI:** 10.7150/ijms.78766

**Published:** 2023-04-23

**Authors:** Lei Yang, LingFang Xu, Qiao Guo, Bing Deng, Yang Hong, LiangLiang Wang, YaLin Wang, DePeng Jiang, Hong Ren

**Affiliations:** 1 Department of Respiratory Medicine, the Second Affiliated Hospital, Chongqing Medical University, Chongqing, China.; 2 Key Laboratory of Molecular Biology for Infectious Diseases (Ministry of Education), Institute for Viral Hepatitis, Department of Infectious Diseases, the Second Affiliated Hospital, Chongqing Medical University, Chongqing, China.; 3 Department of Endocrinology and Metabolism, The Affiliated Hospital of Qingdao University, Qingdao, China.; 4 Department of General Medicine, the Second Affiliated Hospital, Chongqing Medical University, Chongqing, China.

## Abstract

**Purpose:** The effectiveness of inactivated vaccines against acute respiratory syndrome coronavirus 2 (SARS‑CoV‑2), the causative agent of coronavirus disease 2019 (COVID-19), has become a global concern. Hence, the aim of this study was to evaluate vaccine safety and to assess immune responses in individuals with chronic respiratory disease (CRD) following a two-dose vaccination.

**Methods:** The study cohort included 191 participants (112 adult CRD patients and 79 healthy controls [HCs]) at least 21 (range, 21-159) days after a second vaccination. Frequencies of memory B cells (MBCs) subsets and titers of SARS-CoV-2 neutralizing antibodies (NAbs) and anti-receptor binding domain (RBD) IgG antibodies (Abs) were analyzed.

**Results:** As compared to the HCs, CRD patients had lower seropositivity rates and titers of both anti-RBD IgG Abs and NAbs, in addition to lower frequencies of RBD-specific MBCs (all, *p* < 0.05). At 3 months, CRD patients had lower seropositivity rates and titers of anti-RBD IgG Abs than the HCs (*p* < 0.05). For CoronaVac, the seropositivity rates of both Abs were lower in patients with old pulmonary tuberculosis than HCs. For BBIBP-CorV, the seropositivity rates of CoV-2 NAbs were lower in patients with chronic obstructive pulmonary disease than HCs (all, *p* < 0.05). Meanwhile, there was no significant difference in overall adverse events between the CRD patients and HCs. Univariate and multivariate analyses identified the time interval following a second vaccination as a risk factor for the production of anti-RBD IgG Abs and CoV-2 NAbs, while the CoronaVac had a positive effect on the titers of both Abs. Female was identified as a protective factor for CoV-2 NAb levels.

**Conclusion:** Inactivated COVID-19 vaccines were safe and well tolerated by CRD patients but resulted in lower Ab responses and the frequencies of RBD-specific MBCs. Therefore, CRD patients should be prioritized for booster vaccinations.

## Introduction

To date, there have been more than 600 million confirmed cases of infection with severe acute respiratory syndrome coronavirus 2 (SARS‑CoV‑2), the causative agent of coronavirus disease 2019 (COVID-19). Vaccination against COVID-19 is essential to prevent COVID-19 and has altered the trajectory of the pandemic by averting an estimated 19.8 million deaths 1. Risk factors for severe forms of COVID-19 include a history of chronic respiratory disease (CRD), such as chronic bronchitis (CB), chronic obstructive pulmonary disease (COPD), and old pulmonary tuberculosis (OPTB) 2-4.

Patients with COPD reportedly develop a sustained antibody (Ab) response after two doses of vaccination, but at lower levels than healthy controls (HCs) 5. However, Ab levels do not significantly decrease in patients with a history of CRD 6. Currently, changes to Ab levels in CRD patients following vaccination remain unclear, thus additional studies are required.

In addition to the generation of antigen-producing plasma cells, vaccination also induces the differentiation of memory B cells (MBCs), which help to protect against re-exposure to a pathogen 7. MBCs rapidly multiply and terminally differentiate into Ab-secreting cells 8, 9. However, there is a lack of data on clonal expansion of MBCs in CRD patients.

Therefore, the aim of the present study was to assess the titers of MBCs, neutralizing antibodies (NAbs) against SARS-COV-2, and Abs against the receptor-binding domain (RBD) of immunoglobulin G (IgG) in CRD patients after vaccination with an inactivated COVID-19 vaccine.

## Methods

### Study approval and patient consent

The study protocol was approved by the Ethics Committee of the Second Affiliated Hospital of Chongqing Medical University and conducted in accordance with the ethical principles for medical research involving human subjects described in the Declaration of Helsinki. Prior to inclusion in this study, written informed consent was obtained from all subjects. This study is registered with ClinicalTrials.gov (NCT05043246).

### Study cohort

The study cohort included 112 patients with CRDs (43 with COPD, 35 with OPTB, and 34 with CB) and 79 healthy volunteers recruited from the Second Affiliated Hospital of Chongqing Medical University (Chongqing, China). All patients with COPD received low-dose β-agonists/corticosteroids with no recent acute attack and had a ratio of forced expiratory volume in 1 second/slow vital capacity of < 0.7. All patients with CB had symptoms of cough and excess sputum production for at least 3 months and up to 2 years or more. All patients with a prior history of tuberculosis infection had no symptoms of hot flushes, night sweats, or fibrosis/calcification on the lungs, and sputum samples were free of tubercle bacilli. The inclusion criteria for all participants were (i) vaccination with two doses of the BBIBP COVID-19 vaccine (BBIBP-CorV; China National Pharmaceutical Group Corporation, Beijing, China) or CoronaVac vaccine (Sinovac Biotech, Beijing, China) (ii) age ≥ 18 years, (iii) no prior history of COVID-19, (iv) normal immune function, and (v) no current pregnancy.

To standardize the timing of vaccination, “1 month” was defined as 21-45 days (46 CRD patients and 42 HCs), “2 months” as 46-75 days (26 CRD patients and 18 HCs), and “3 months” as 76-105 days (29 CRD patients and 15 HCs).

### Ab responses

Plasma levels of RBD IgG Abs and NAbs were detected with the MAGLUMI 2000 Chemiluminescence Immunoassay System (Snibe, Shenzhen, China). Cut-off values for seropositivity of RBD IgG Abs and NAbs were >1 AU/mL and >0.15 μg/mL, respectively.

### Identification of MBCs

Fresh peripheral blood samples were collected and centrifuged through a Ficoll density gradient (Histopaque; Sigma-Aldrich Corporation, St Louis, MO, USA). Brilliant Violet 421™ Streptavidin-conjugated Abs (BioLegend, San Diego, CA, USA) and biotinylated Abs against the RBD of the SARS-CoV-2 spike protein (Sino Biological, Beijing, China) at a molar ratio of 4:1 were used for identification of the MBCs. For flow cytometry (Beckman Coulter, Inc., Brea, CA, USA), the cells were washed with phosphate-buffered saline, suspended staining buffer containing 2% fetal bovine serum, and probed with Abs against IgG, IgM, cluster of differentiation 3 (CD3), CD19, CD21, and CD27 (all, Biolegend). The data were examined using FlowJo software (version 10.0.7; FlowJo, LLC, Ashland, OR, USA). Abs against CD3, CD19, CD21, and CD27 were used to differentiate MBCs into subsets of RBD-specific MBCs (CD3^-^/CD19^+^/RBD^+^/CD27^+^), RBD^+^ atypical MBCs (CD3^-^/CD19^+^/RBD^+^/CD21^-^/CD27^-^), RBD^+^ intermediate MBCs (CD3^-^/CD19^+^/RBD^+^/CD21^+^/CD27^-^), RBD+ activated MBCs (CD3^-^/CD19^+^/RBD^+^/CD21^-^/CD27^+^), and RBD^+^ resting MBCs (CD3^-^/CD19^+^/RBD^+^/CD21^+^/CD27^+^).

### Statistical analysis

The chi-square test or Fisher exact test was used to compare categorical variables. For continuous variables, the Student's *t*-test or the Mann-Whitney U test was used for comparisons of two groups, and the Kruskal-Wallis test for three or more groups. All results of multiple comparisons were corrected using Dunn's multiple comparisons test. Univariate and multivariate linear regression analyses were used to identify risk and protective factors associated with Ab titers. All outcomes were adjusted. Data analysis was conducted using IBM SPSS Statistics for Windows, version 26.0 (IBM Corporation, Armonk, NY, USA). Figures were generated with GraphPad Prism software (version 9.2.0; GraphPad Software, Inc., San Diego, CA, USA). A probability (*p*) value < 0.05 was considered statistically significant.

## Results

### Population Characteristics

The study cohort included a total of 191 participants. As shown in **Table 1**, comparisons between the CRD patients and HCs found no significant differences in median age, percentage of males, mean body mass index, vaccine type, median number of days after 2^nd^ vaccination, results of routine blood tests (white blood cells, hemoglobin, lymphocytes, and platelets), and liver function markers (aspartate transaminase and alanine aminotransferase).

## Safety

Following immunization, all adverse events (AEs) within the first week were categorized using the criteria of the China Medical and Drug Administration (2019 edition). There were no significant differences in overall, local, and systemic AEs between the CRD patients and HCs. Notably, there were no severe AEs (Table 2).

### Humoral immune responses to inactivated SARS-CoV-2 vaccines in CRD patients

Overall, when compared to HCs, CRD patients had significantly reduced seropositivity rates for anti-RBD IgG Ab and CoV-2 NAb(**Table 3**).

**Figure 1:**
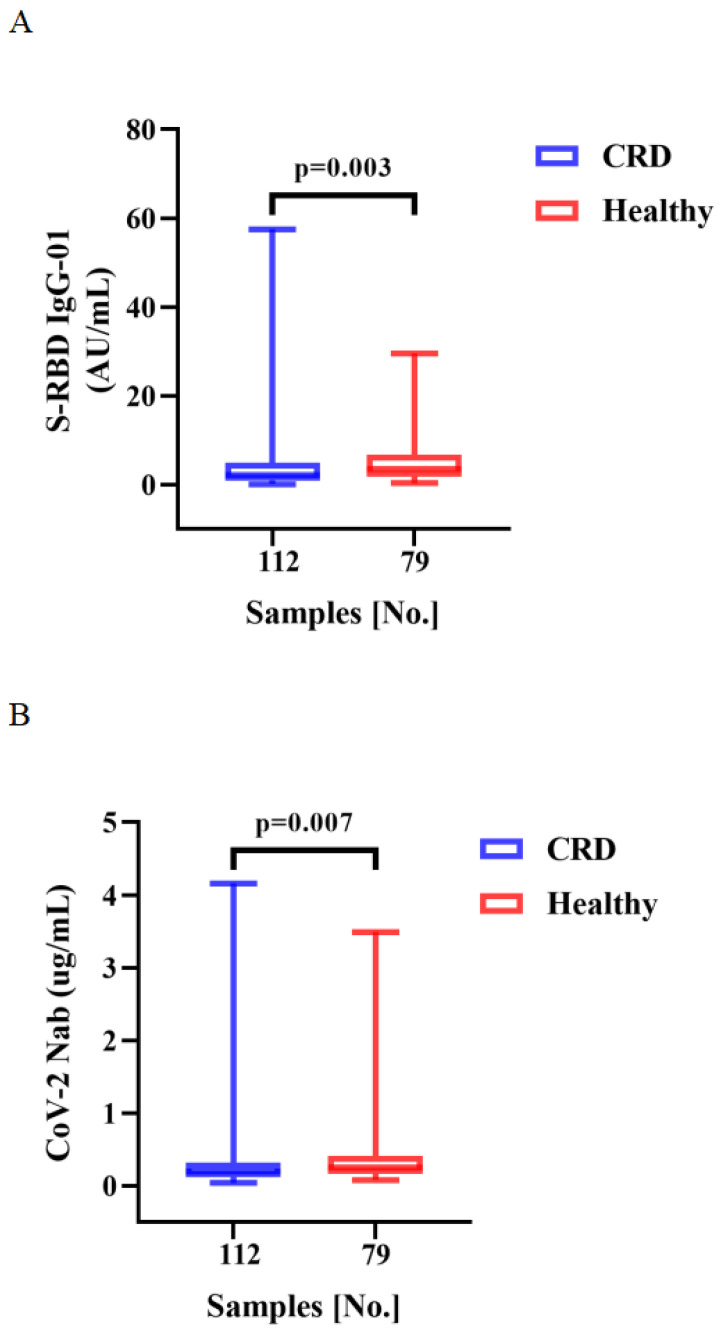

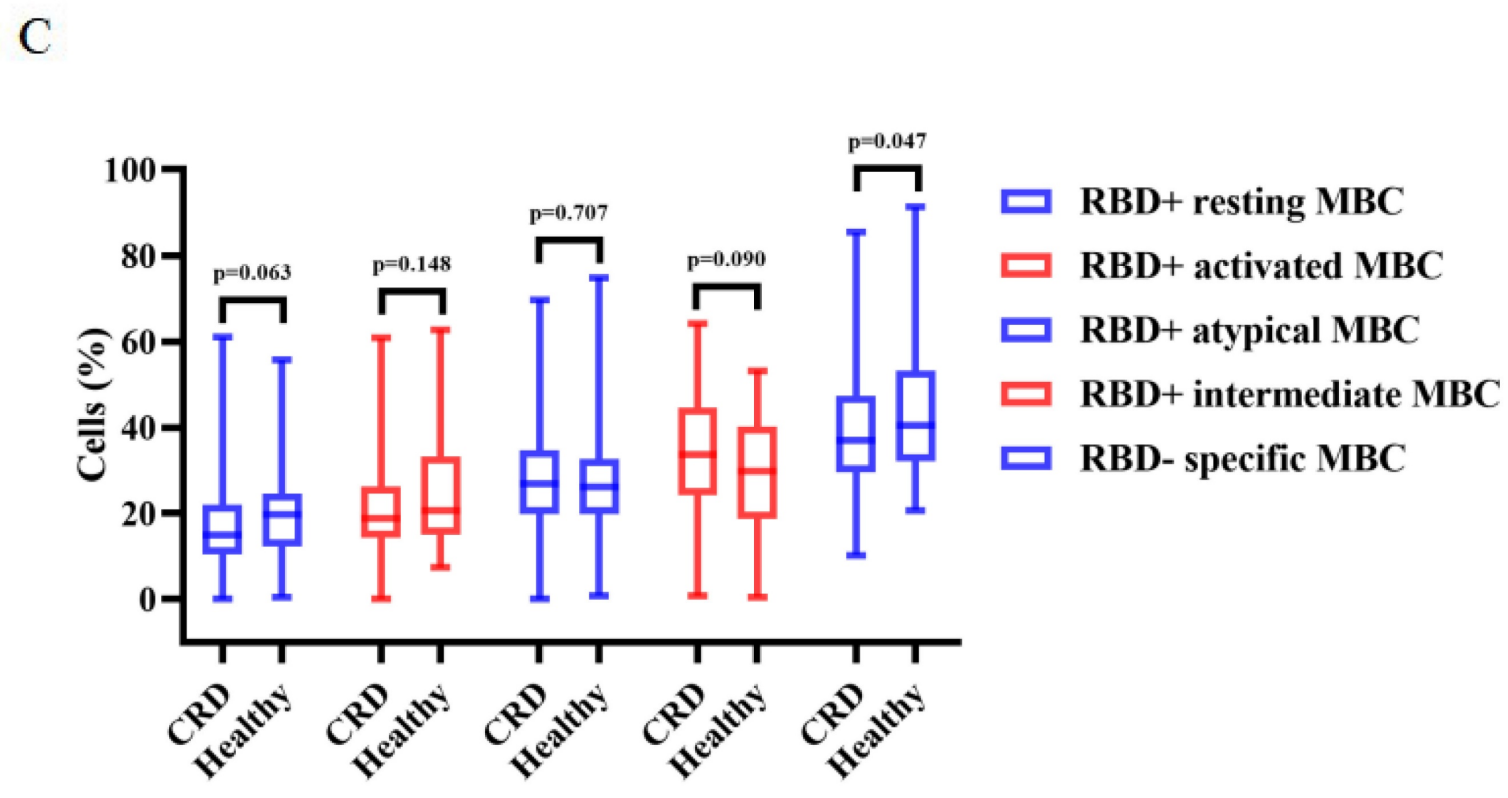
** A-C**. Humoral immune responses to inactivated vaccines in CRD patients.

Vaccination induced production of anti-RBD IgG Abs and CoV-2 NAbs in all CRD patients and HCs. As compared to the HCs, CRD patients had significantly lower titers of anti-RBD IgG (median [IQR]: 2.10 [0.86-4.855] vs. 3.45 [1.84-6.79], respectively, *p* = 0.003) and SARS-CoV-2 NAbs (median [IQR]: 0.20 [0.12-0.32] vs. 0.25 [0.16-0.41], *p* = 0.007) (**Figure 1A, B**). However, the frequencies of RBD-specific MBCs were lower in CRD patients than the HCs (median [IQR]: 37.05 [29.43-47.30] vs. 40.4 [32.00-51.20], *p* = 0.047), while there was no significant difference in the frequencies of other MBC subsets (**Figure 1C**).

Between CRD patients and HCs, **Figure 1A, B**, the titers of anti-RBD IgG Abs and CoV-2 NAbs. **Figure 1C** show, the frequencies of RBD^+^ resting MBCs, RBD^+^ activated MBCs, RBD^+^ atypical MBCs, RBD^+^ intermediate MBCs, and RBD-specific memory B cells (MBCs). The Mann-Whitney U test was employed to compare the Ab titers and the frequencies of MBCs.

### Humoral immune responses to inactivated SARS-CoV-2 vaccines in CRD patients over time

Between CRD patients and HCs, there was a significant difference in the seropositivity rates of anti-RBD IgG Ab after 3 months (**Table 4**).

**Figure 2:**
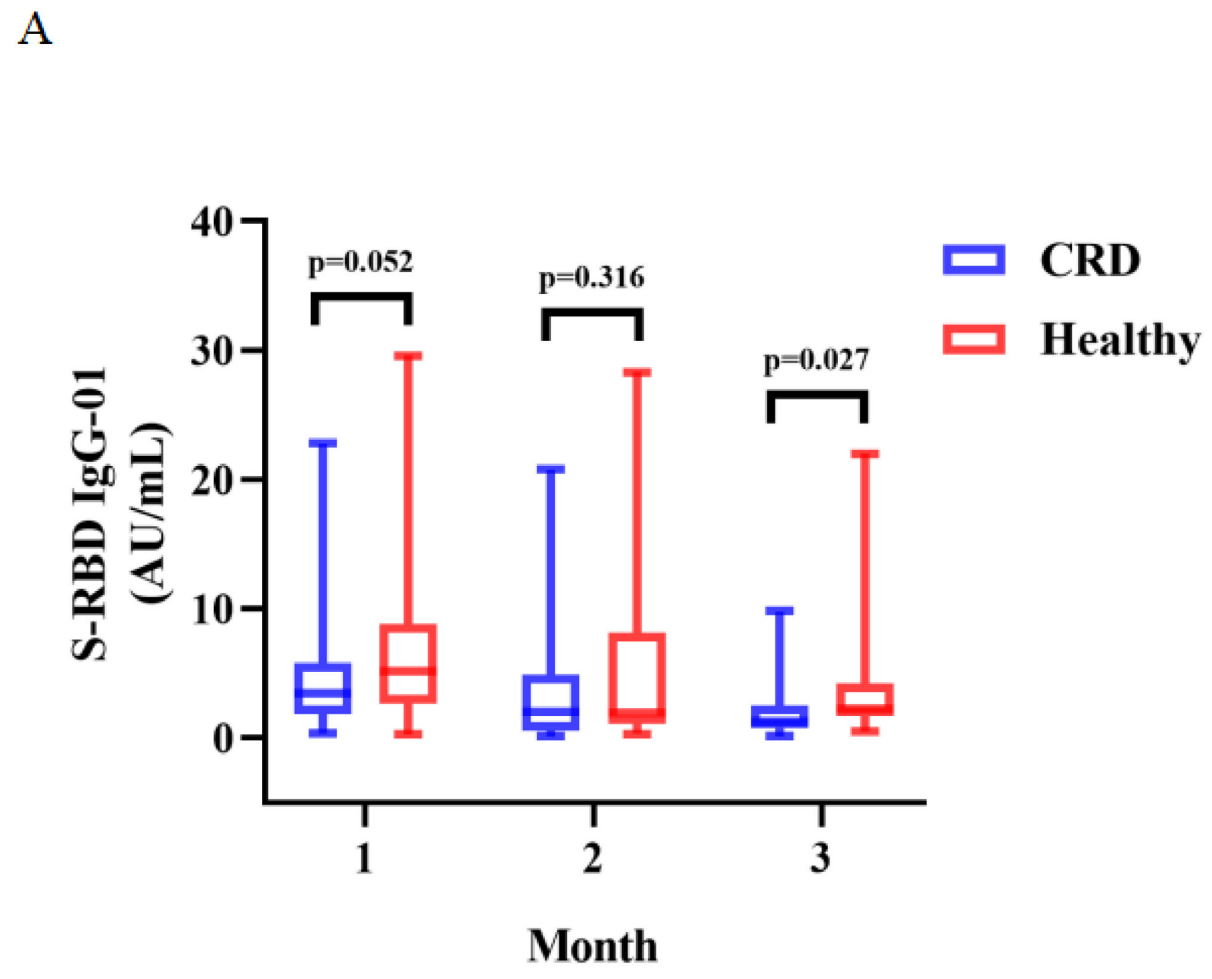

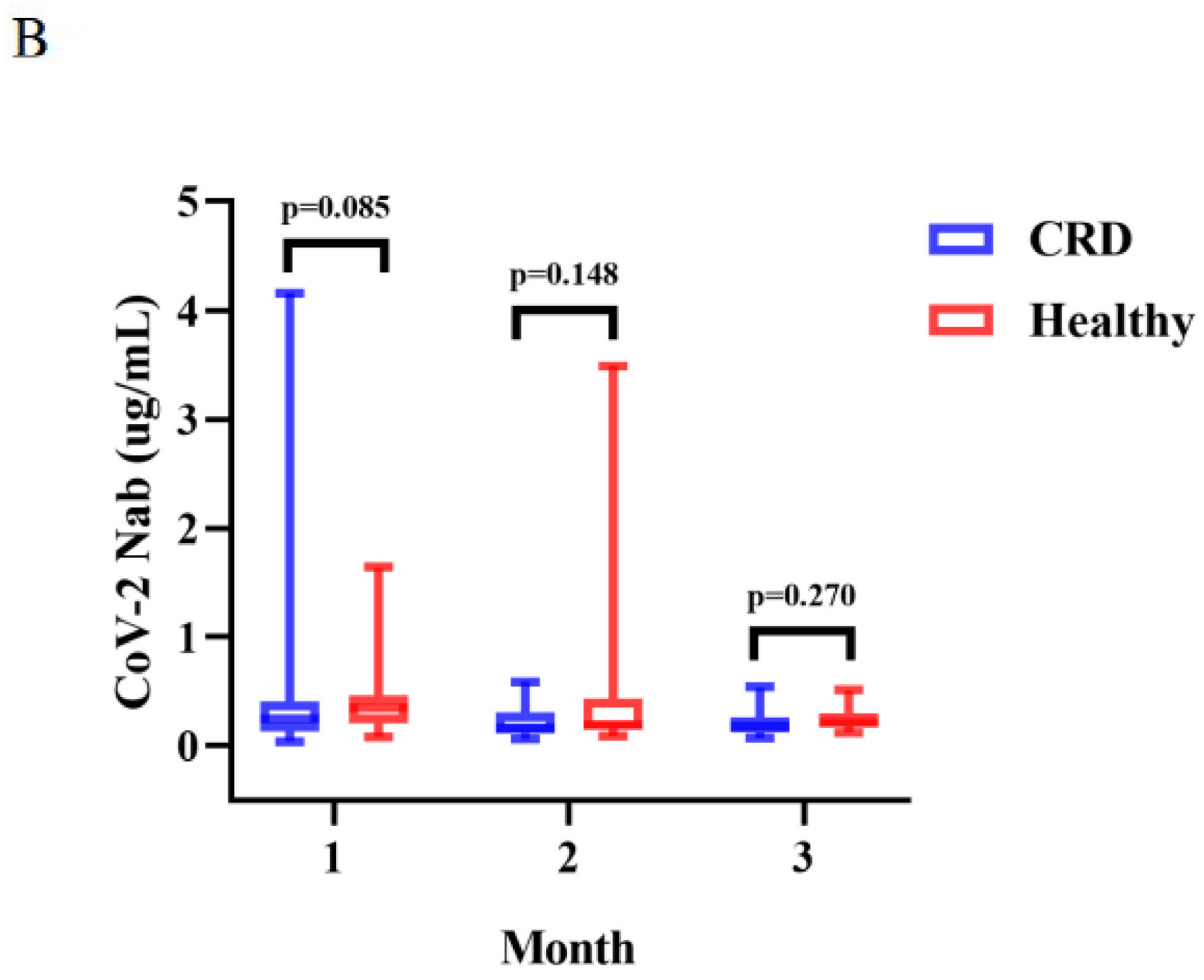
** A-B**. Antibody responses to inactivated vaccines in CRD at 1, 2, 3 month.

Between CRD patients and HCs, there was a significant difference in the titers of anti-RBD IgG at 3 months (median [IQR]: 1.29 [0.75-2.50] vs. 2.24 [1.68-4.22], *p* = 0.027) (**Figure 2A**). The titers of anti-RBD IgG Abs were slightly lower in CRD patients at 1 and 2 months as compared to HCs, as well as CoV-2 NAbs at 1, 2, and 3 months, although these differences were not significant (**Figure 2A** and** B**, respectively**)**. In addition, the frequencies of RBD-specific MBCs was somewhat lower in CRD patients than the HCs at each time point, although this difference was not statistically significant (**Supplementary Figure 1A-E**).

The titers of anti-RBD IgG Abs and CoV-2 NAbs were measured in CRD patients and HCs at 1, 2, and 3 months were shown in **Figure 2A-B**. The Mann-Whitney U test was employed to compare the Ab titers.

### Comparison of Humoral immune responses to CoronaVac and BBBIBP-CorV

As a result, the Corona Vac had higher seropositivity rates of both Abs than BBIBP-CorV in CRD patients (**Table 5**), and the CRD patients had higher seropositivity rates of anti-RBD IgG Ab than HCs (Corona Vac) (**Table 6**).

**Figure 3:**
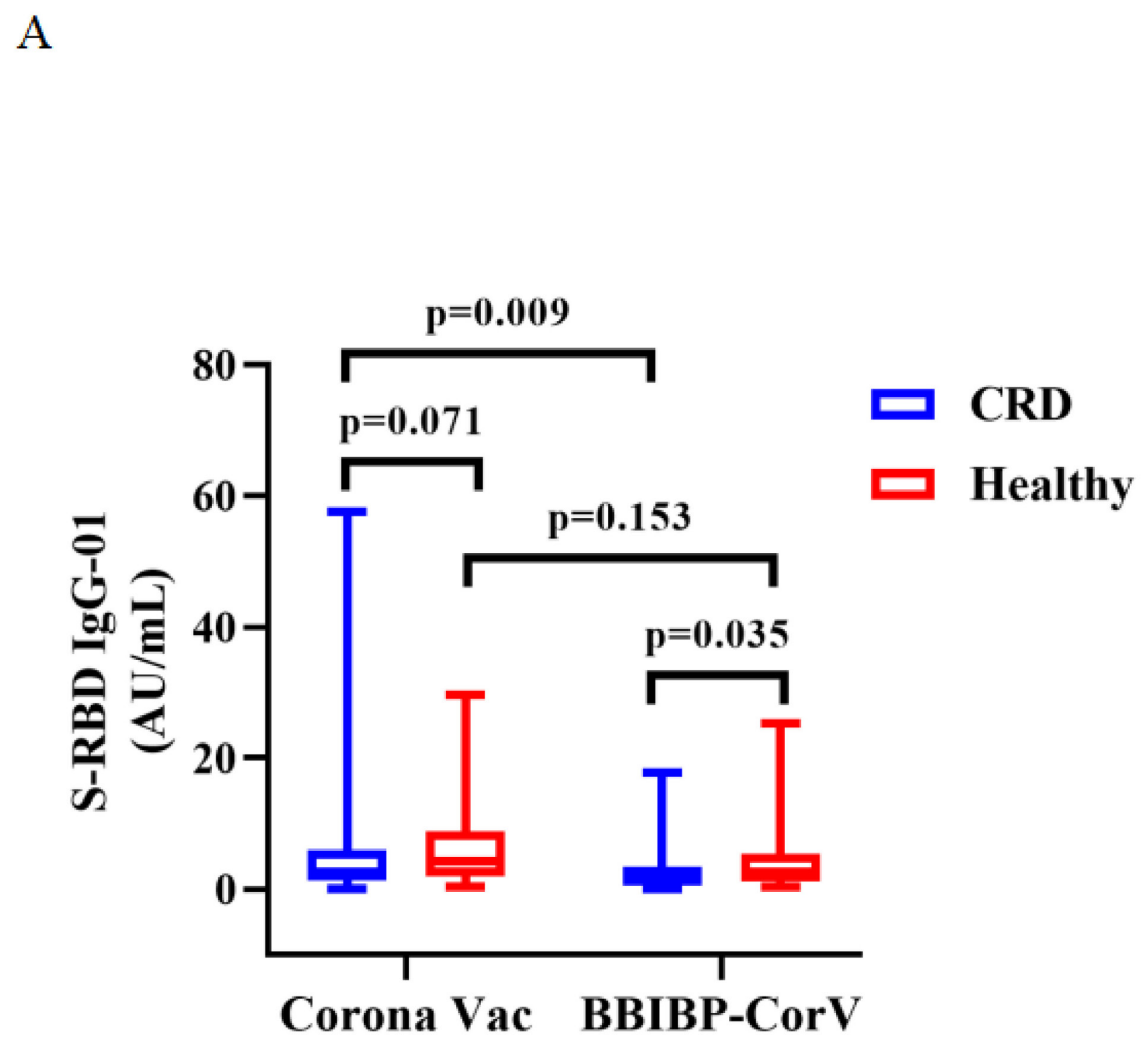

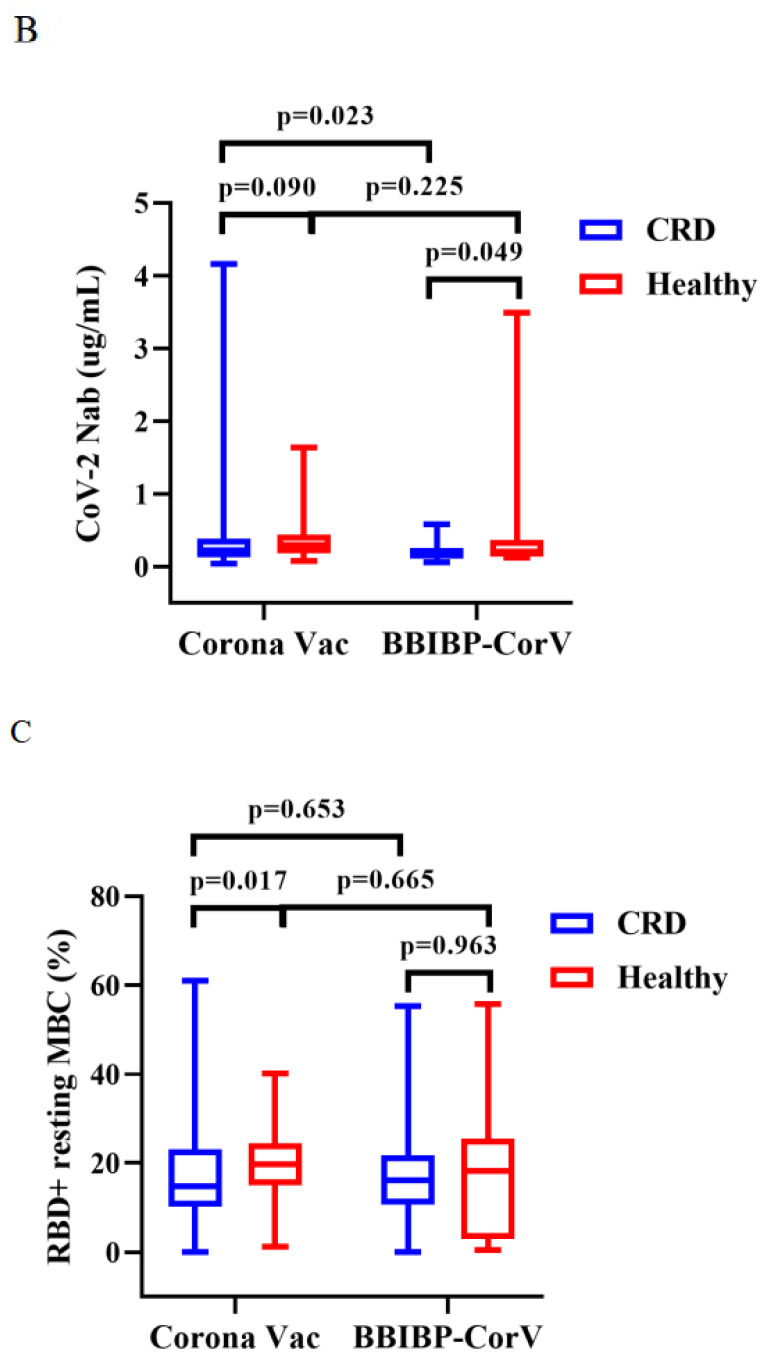

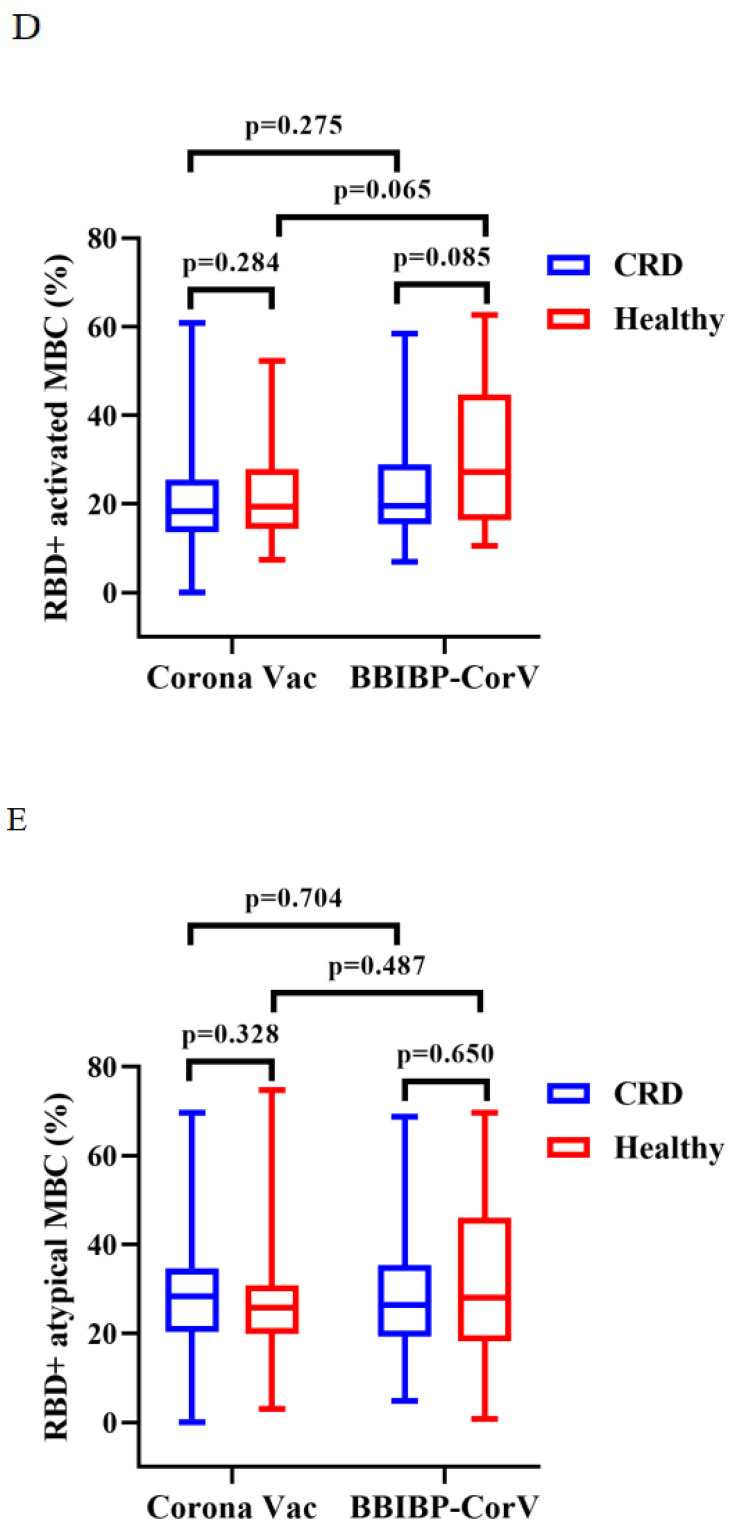

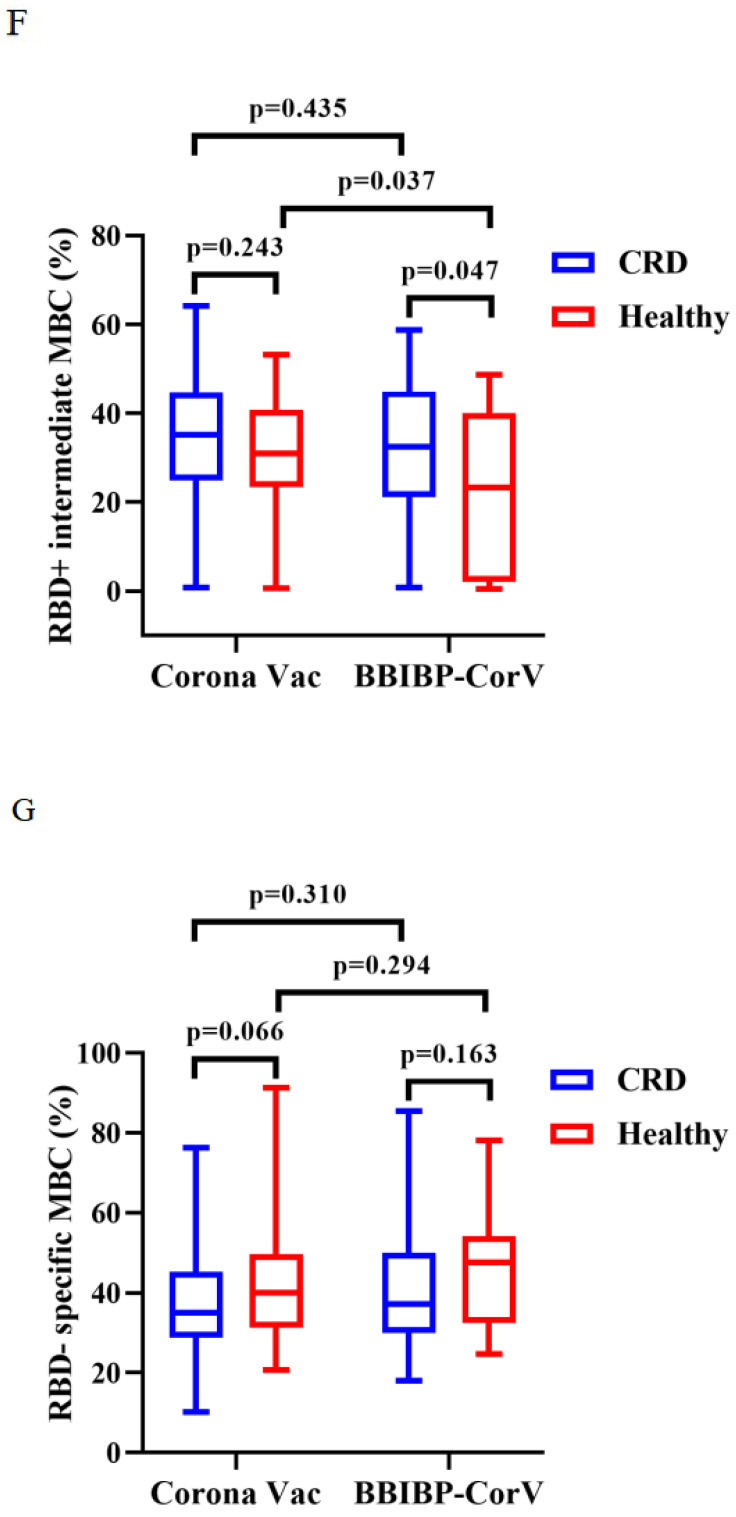
** A-G**. Humoral immune responses to inactivated vaccines in CRD patients and HCs by vaccine type.

Titers of both Abs induced by CoronaVac were higher than those by BBIBP-CorV in CRD patients (median [IQR]: 2.67 [1.29-5.91] vs. 1.54 [0.57-3.37], *p* = 0.009, and 0.22 [0.13-0.38] vs. 0.15 [0.12-0.26], *p* = 0.023), while the titers of both Abs induced by BBIBP-CorV were higher in HCs than CRD patients (median [IQR]: 1.54 [0.57-3.37] vs. 2.64 [1.10-5.30], *p* = 0.035, and 0.15 [0.12-0.26] vs. 0.21 [0.14-0.36], *p* = 0.049) (**Figure 3A, B**). The frequencies of RBD^+^ resting MBCs was lower in the HCs than CRD patients after vaccination with CoronaVac (median [IQR]: 14.70 [10.20-23.00] vs. 19.70 [14.95-24.40], *p* = 0.017), while the frequencies RBD^+^ intermediate MBCs was higher after vaccination with BBIBP-CorV (median [IQR]: 32.40 [21.10-44.90] vs. 23.30 [2.09-40.03], *p* = 0.047). Meanwhile, the frequencies of RBD^+^ intermediate MBCs was higher with the CoronaVac than the BBIBP-CorV in the HCs (median [IQR]: 30.90 [23.40-40.70] vs. 23.30 [2.09-40.03], *p* = 0.037), while there was no significant difference in the frequencies of other MBC subsets (**Figure 3C-G**).

The titers of anti-RBD IgG Abs and CoV-2 NAbs in CRD patients and HCs (corona vac and BBIBP-corV) were shown in **Figure 3A, B**. The frequencies of RBD^+^ resting MBCs, RBD^+^ activated MBCs, RBD^+^ atypical MBCs, RBD^+^ intermediate MBCs and RBD-specific memory B cells (MBCs) in CRD patients and HCs (corona vac and BBIBP-corV) were shown in **Figure 3C-G**. The Mann-Whitney U test was used to compare the Ab titers and the frequencies of MBCs.

### Humoral immune responses to inactivated SARS-CoV-2 vaccines in CRD subgroups

 The seropositivity rates of both Abs (Corona Vac) were lower in OPTB patients than HCs, and the seropositivity rates of CoV-2 NAb(BBIBP-CorV) were lower in COPD patients than HCs, the seropositivity rates of CoV-2 NAb(Corona Vac) were significant different in CB, OPTB and COPD patients(**Table 7**).

There was no significant difference in the titers of both Abs after vaccination with Corona Vac or BBIBP-CorV in each CB, OPTB, COPD patients compared with HCs (all, *p* > 0.05). The titers of both Abs were similar in CB, OPTB, COPD patients after vaccination with Corona Vac(6.33[2.02-9.07] vs. 2.27[0.80-4.69] vs. 2.39[1.23-5.20], *p* = 0.103 and 0.32[0.20-0.49] vs. 0.22[0.11-0.43] vs. 0.19[0.13-0.31], *p* = 0.055, respectively), at the same time, the titers of both Abs were no significant difference in CB, OPTB, COPD patients after vaccination with BBIBP-CorV(1.54[0.67-2.25] vs. 1.99[0.57-3.87] vs. 0.89[0.51-4.91], *p =* 0.941 and 0.17[0.12-0.21] vs. 0.18[0.09-0.32] vs. 0.15[0.12-0.30], *p* = 0.947, respectively).

**Figure 4:**
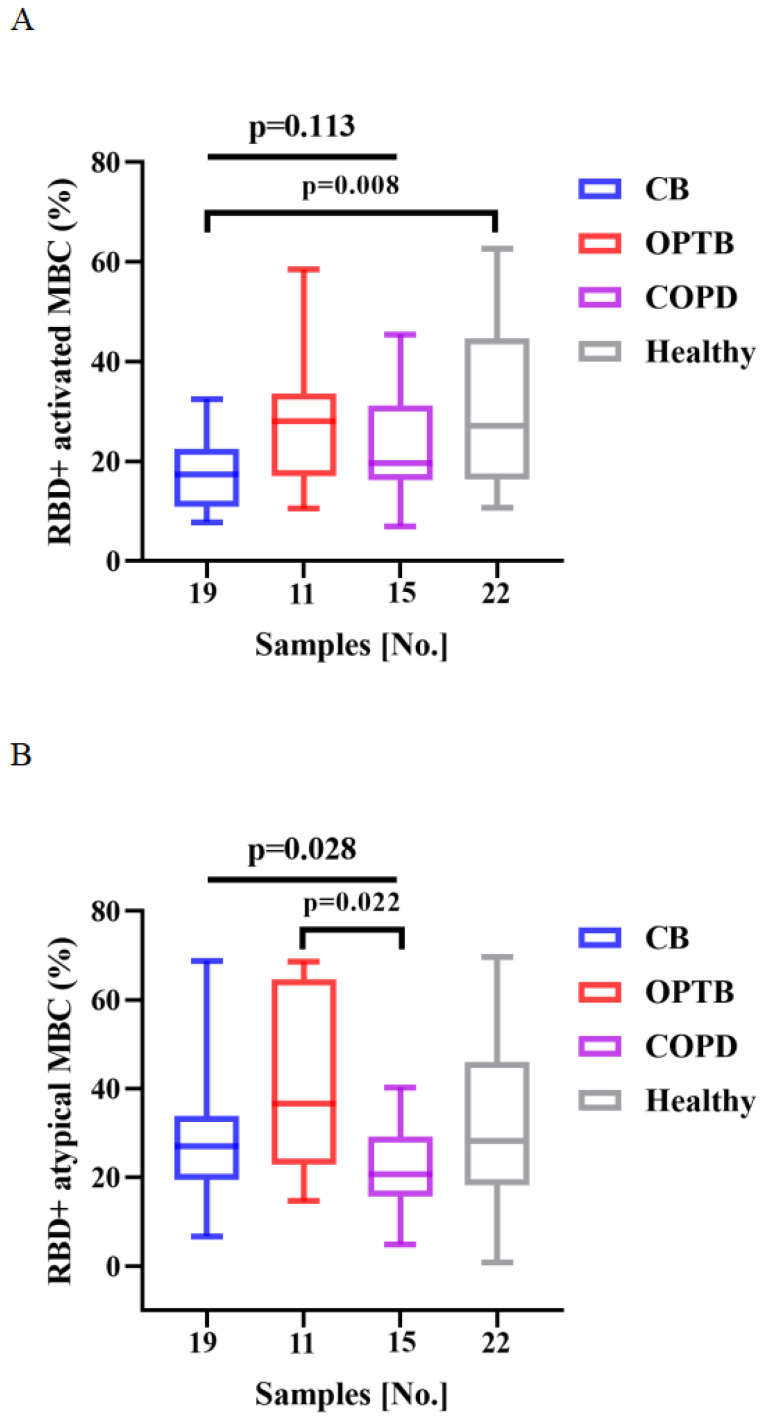

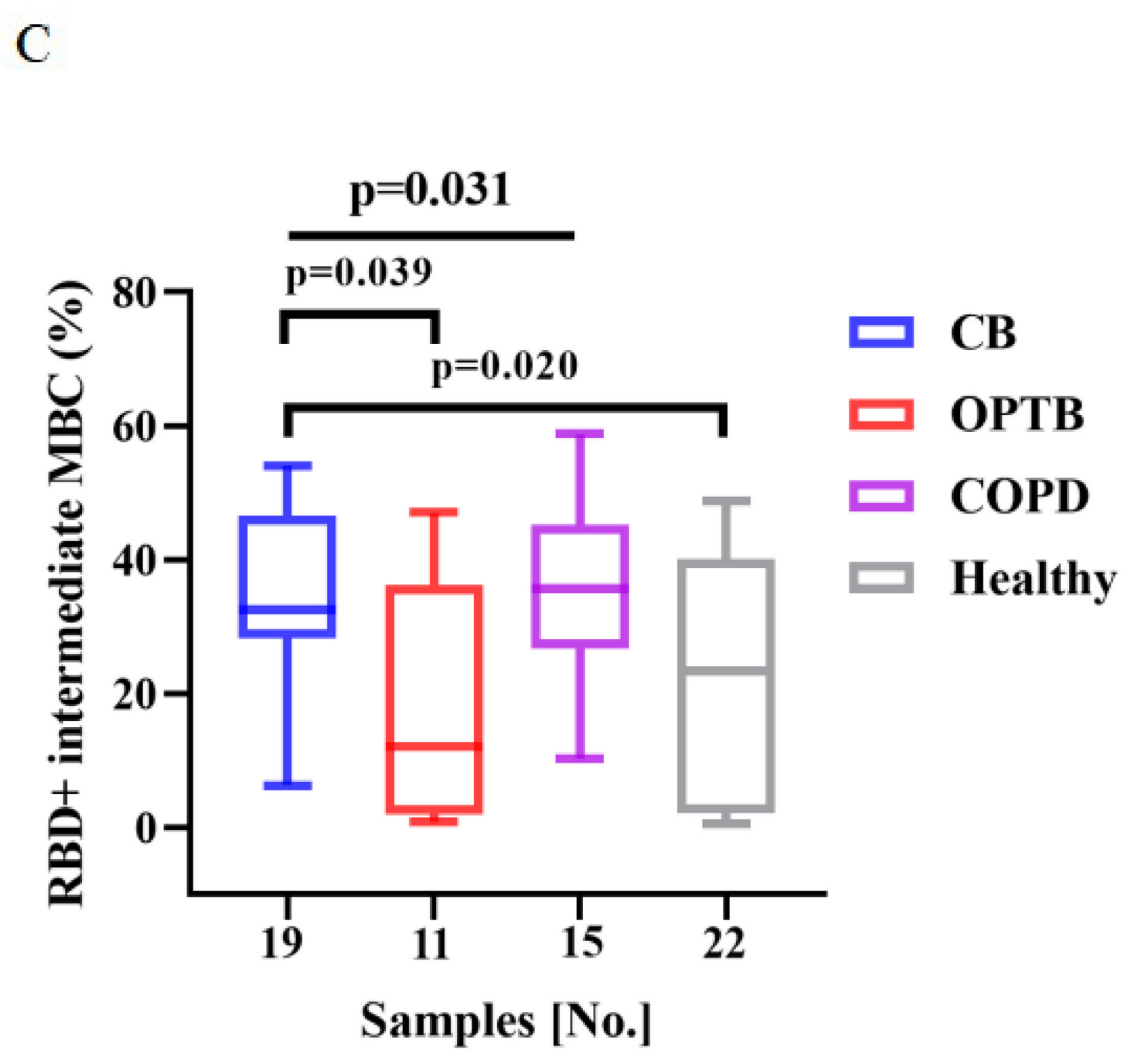
** A-C**. Frequencies of MBC subsets in response to inactivated vaccines in CRD subgroups.

The frequencies of RBD^+^ activated MBCs was lower and that of RBD^+^ intermediate MBCs was higher in CB patients than HCs after vaccination with BBIBP-CorV (*p* = 0.008 and 0.020, respectively) (**Figure 4A** and** C**). There were significant differences in the frequencies of RBD^+^ atypical MBCs and RBD^+^ intermediate MBCs among the CB, OPTB, and COPD patients after vaccination with BBIBP-CorV, while the proportion of RBD^+^ atypical MBCs was lower in COPD patients than OPTB patients and the frequencies of RBD^+^ intermediate MBCs was higher in CB patients than OPTB patients (**Figure 4B** and** C**).

The frequencies of RBD^+^ activated MBCs and RBD^+^ intermediate MBCs of the CB, OPTB, and COPD patients and HCs after vaccination with BBIBP-CorV are shown in** Figure 4A** and** C**. The frequencies of RBD^+^ atypical MBCs and RBD^+^ intermediate MBCs in the CB, OPTB, and COPD patients after vaccination with BBIBP-CorV are shown in **Figure 4B** and** C**. The Kruskal-Wallis test and Dunn's multiple comparisons test were employed to compare the frequencies of MBCs.

### Humoral immune responses to inactivated SARS-CoV-2 vaccines by age

As a result, there were no significant difference of the seropositivity rates of both Abs in CRD patients aged ≥ 60 and < 60 years, which is consistent with HCs (**Table 8**).

**Figure 5:**
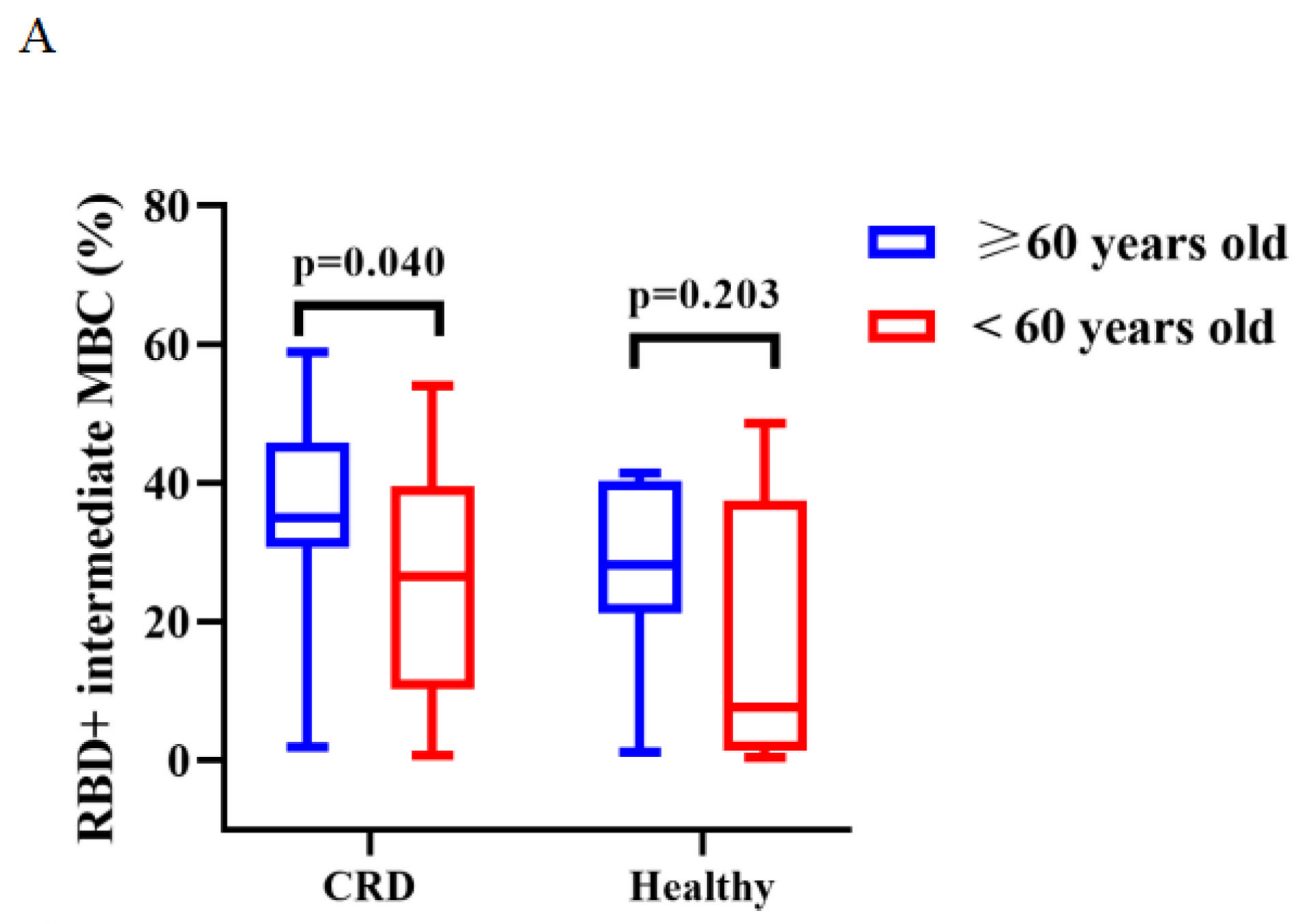

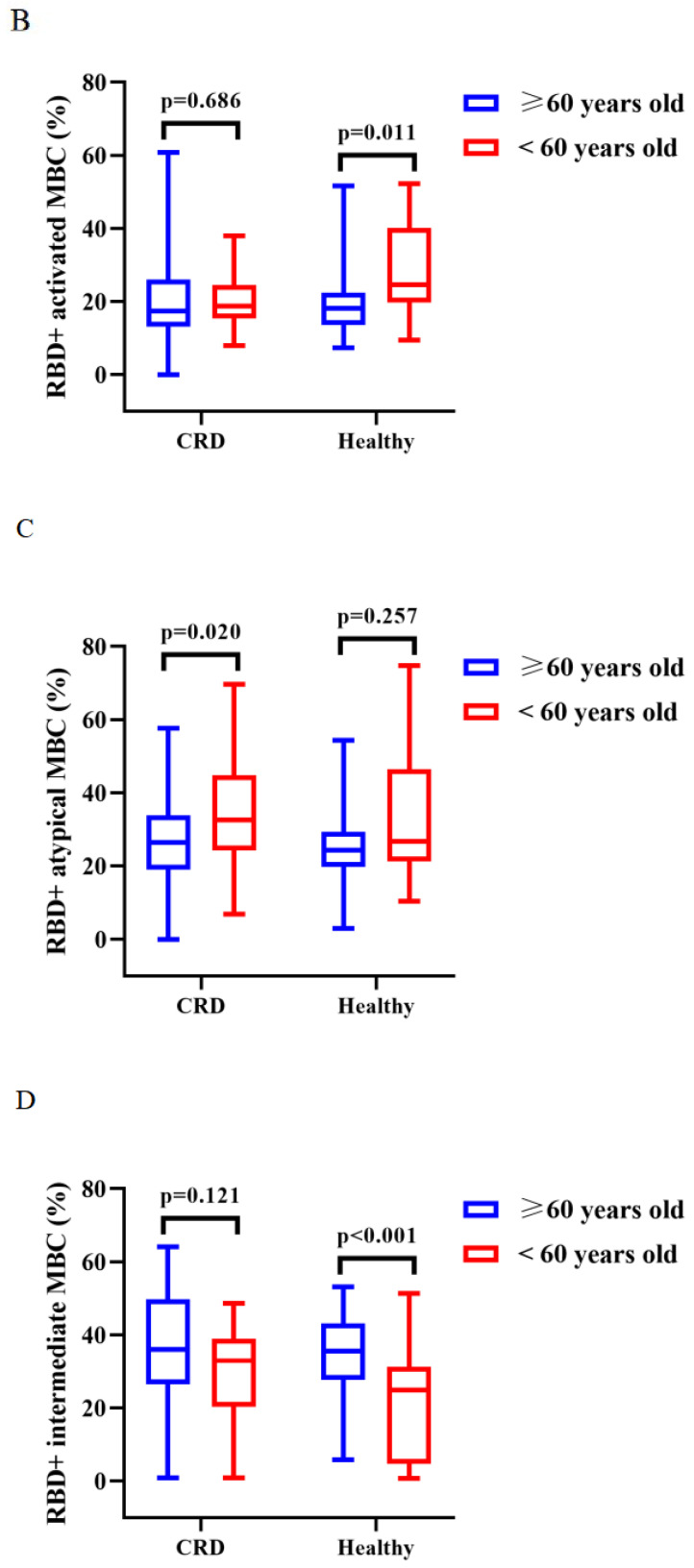
** A-D**. Frequencies of MBC subsets in response to inactivated vaccines aged ≥60 and <60 years.

The titers of anti-RBD IgG Abs and CoV-2 NAbs were similar in CRD patients and HCs aged ≥ 60 and < 60 years after vaccination with CoronaVac (**Supplementary Figure 3A, B**) and BBIBP-CorV (**Supplementary Figure 3C, D**). After vaccination with CoronaVac, as compared to HCs aged ≥60 years, those aged <60 years had greater frequencies of RBD^+^ activated MBCs (median [IQR]: 18.05 [13.48-22.45] vs. 24.50 [19.65-40.20], *p* = 0.011) and lower frequencies of RBD^+^ intermediate MBCs (34.82 [95% CI = 31.29-38.34] vs. 21.04 [95% CI = 13.57-28.51], *p* < 0.001), while CRD patients aged ≥60 vs. <60 years had lower frequencies of RBD^+^ intermediate MBCs (35.09 [95% CI = 29.61-40.57] vs. 25.33 [95% CI = 16.98-33.68], *p* = 0.040) and RBD^+^ atypical MBCs (26.83 [95% CI = 23.28-30.38] vs. 35.33 [95% CI = 27.90-42.76], *p* = 0.020) (BBIBP-CorV: **Figure 5A**; CoronaVac **Figure 5B-D**), while there were no significant differences in the frequencies of the others MBC subsets (BBIBP-CorV: **Supplementary Figure 3E-H;** CoronaVac: **Supplementary Figure 3I-J**).

**Figure 5A** (BBIBP-CorV) and** B-D** (Corona Vac) shows, the frequencies of RBD^+^ activated MBCs, RBD^+^ atypical MBCs and RBD^+^ intermediate MBCs in CRD patients and HCs aged ≥60 and <60 years. The T test, Mann-Whitney U test were used to compare the Ab titers and the frequencies of MBCs.

Other confounding factors are listed in **Tables 9** and **10**. The duration of time following the second vaccination was identified as a risk factor for serum levels of anti-RBD IgG Abs and CoV-2 NAbs, while the type of vaccine (CoronaVac) had a protective impact on Ab response and female was a protective factor for CoV-2 NAb levels.

## Discussion

In this prospective observational trial, Ab levels and responses of MBC subsets were measured to assess the efficacy and safety of inactivated SARS-CoV-2 vaccines.

The results clarified that inactivated vaccines were safe and well tolerated in CRD patients. The most prevalent local and systemic AEs were pain and exhaustion, respectively, while there were no severe AEs, such as myocardial infarction and thromboembolic events 10, 11. As compared to earlier large-scale studies 12, 13, the likelihood of AEs following immunization in these patients was significantly reduced.

CRD patients had significantly lower serum Ab responses after the second vaccination, but were still detected after 6 months, consistent with previous studies 5, 15. A previous study indicated that vaccine efficacy for non-immunocompromised patients with chronic diseases were similar between risk groups 14. In addition, the seroconversion rate was lower for CRD patients as compared to the HCs, in agreement with a previously published study 16. Overall, CRD patients had low immunogenicity to inactivated COVID-19 vaccines.

Anti-RBD IgG Ab and NAb levels were higher in the HCs as compared to the CRD patients for BBIBP-CorV, although not always significant for CoronaVac, possibly due to the relatively small study cohort. Interestingly, the response to BBIBP-CorV was lower in CRD patients than for CoronaVac. The results of linear regression analysis identified CoronaVac as a protective factor that promotes Ab responses, consistent with the findings of a previous study 17, but not for the HCs. These results suggest that BBIBP-CorV should not be administered to CRD patients. However, the lower titers of anti-RBD IgG Abs and NAbs of the CRD patients who received BBIBP-CorV may be due to the higher proportion of males, longer period between vaccinations, or the greater proportion of CB patients.

In this study, OPTB patients had lower seropositivity rates for both Abs following vaccination with CoronaVac, while COPD patients had lower seropositivity rates of CoV-2 NAbs following vaccination with BBIBP-CorV. However, this difference was not significant in other subgroups, possibly due to the higher proportion of males or the longer period between vaccinations and sample collection. The seropositivity rates of CoV-2 NAbs were significant difference after vaccination with CoronaVac among CB, OPTB, COPD patients, it might also be that OPTB patients have the higher proportion of males.

The germinal center (GC) and an extra-follicular GC-independent mechanism can both produce MBCs 18, 19. The GC is the primary source of class-switching and significant alterations in somatic Abs 18, 20. Among the MBC subsets, the frequencies of IgG^+^ RBD-specific MBCs was the lowest 7. MBCs, which differentiate into Ab-secreting cells upon secondary infection, are crucial to maintain long-term humoral immunity 21-23. In a prior investigation, the frequencies of MBCs in peripheral blood were lower in COPD patients than HCs 24. Additionally, counts of late and switched MBCs were decreased in COPD patients 25. After therapy, classical MBCs remained at low levels in patients with tuberculous 26, 27. Previous research has demonstrated that RBD-specific MBCs help to produce Abs 28. In the present study, the frequencies of RBD-specific MBCs were lower in CRD patients than the HCs. Collectively, these data indicate that humoral immunity induced by inactivated COVID-19 vaccinations was compromised in CRD patients.

In this study, age was not associated with Ab responses in CRD patients, either by grouping or mixed-factor analysis. According to earlier studies 29, 30, the Ab response to the ChAdOx1nCoV-19 and mRNA-1273 COVID-19 vaccines in clinical trials was not influenced by age. However, in contrast to previous reports, age >70 years was related to decreased Ab responses in patients with COPD 31 and the immunological response was poor for those aged >55 years after vaccination with CoronaVac 32. However, the sample size was relatively small, thus larger studies are needed.

The time interval after a second dose of the vaccine was linked to poor Ab responses by both univariate and multivariate analyses, which is consistent with known risk factors for suboptimal vaccination responses 33, 34. This finding suggests the need for timely Ab detection and booster doses to maintain a stable Ab response. In a previous study, female was identified as a protective factor for CoV-2 NAb levels 35, consistent with the results of the present study. Higher body mass index was related to lower Ab titers 36, but this finding was not confirmed by univariate or multivariate analysis, possibly due to the relatively small sample size.

There were some limitations to this cross-sectional investigation that should be addressed. First, the study participants were recruited from a single center. Second, T cell levels were not analyzed. Third, longitudinal analysis was not conducted, as only cross-sectional comparisons were performed. Fourth, the small sample size prohibited assessments of potential effects. Fifth, the results might not be generalizable to other vaccines, settings, and ethnicities. Nonetheless, these findings will help to assess the safety and immunogenicity of the COVID-19 vaccination in CRD patients. Second, responses of two Abs were comprehensively analyzed to assess humoral and cellular immunity to the vaccine. Third, while the type of vaccine was protective for the two Abs, the amount of time since full immunization was confirmed as a risk factor for Ab levels.

In summary, the inactivated COVID-19 vaccines were safe and well tolerated, but the Ab response was modest and the frequencies of RBD-specific MBCs was reduced in CRD patients. Similar results were reported with the anti-RBD IgG response after 3 months. Hence, CRD patients should be prioritized for booster vaccinations.

## Supplementary Material

Supplementary figures.

## Figures and Tables

**Table 1:** General characteristics of CRD patients and HCs.

	CRD patients (n =112)	HCs (n = 79)	p
Age (years),	64.00 (20-84)	64.00 (19-89)	0.895
median (range)			
Sex			
Female	44.6% (50/112)	51.9% (41/79)	0.323
Male	55.4% (62/112)	48.1% (38/79)	
Body mass index,	23.23 (16.60-33.98)	23.92 (17.98-29.42)	0.110
mean (range)			
Vaccine type			
CoronaVac	59.8% (67/112)	72.2% (57/79)	0.079
BBIBP-CorV	40.2% (45/112)	27.8% (22/79)	
Days after 2nd vaccination	54 (21-159)	44 (21-135)	0.071
median (range)			
Routine blood tests			
median (range)			
White blood cell	6.02 (2.67-12.54)	6.09 (2.05-19.08)	0.634
Hemoglobin	137 (89-175)	138 (73-208)	0.952
Lymphocyte	1.70 (0.21-3.64)	1.75 (0.64-3.40)	0.598
mean (range)			
Platelet	197 (94-638)	189 (80-1182)	0.648
Liver function			
median (range)			
Aspartate transaminase	21 (6-118)	20 (3-82)	0.278
Alanine aminotransferase	21 (10-76)	21 (11-60)	0.156

The chi-square test was used for comparisons of categorical variables and the Mann-Whitney U test was used for comparisons of continuous variables.

**Table 2:** AEs associated with vaccination in CRD patients and HCs.

Variable	HTN patients	HCs	p
**Overall AEs**	16(14.3%)	6 (7.6%)	0.154
**Local AEs**			
Pain	4 (3.6%)	3 (3.8%)	0.935
Swelling	1 (0.9%)	/	1.000
Pruritus	1 (0.9%)	1 (1.3%)	0.805
**Systemic AEs**			
Fever	3 (2.7%)	/	1.000
Fatigue	3 (2.7%)	2 (2.5%)	0.950
Drowsiness	2 (1.8%)	1 (1.3%)	0.773
Cough	2 (1.8%)	/	1.000
Dizziness	2 (1.8%)	/	1.000
Gastro spasm	1 (0.9%)	/	1.000
**Grade 3 and 4 AEs**	/	/	

The data are presented as n (%). The chi-square and Fisher's exact tests were used for comparisons.

**Table 3:** Seropositivity rates of both Abs in all CRD patients.

Ab	CRD patients (n = 112)	HCs (n = 79)	*p*
Anti-RBD IgG	69.60% (78/112)	88.80% (70/79)	0.003
CoV-2 NAb	62.50% (70/112)	79.70% (63/79)	0.011

The chi-square test was used for comparisons.

**Table 4:** Seropositivity rates of both Abs in CRD patients at 1, 2, and 3 months.

Ab/NAb	Time after second vaccination (months)	CRD patients	HCs	*p*
Anti-RBD IgG	1	87.00% (40/46)	93.00% (39/42)	0.575
	2	61.50% (16/26)	83.30% (15/18)	0.119
	3	62.10% (18/29)	93.3% (14/15)	0.027
CoV-2 NAb	1	71.7% (33/46)	83.7% (35/42)	0.195
	2	53.8% (14/26)	77.80% (14/18)	0.105
	3	62.10% (18/29)	80.00% (12/15)	0.226

The chi-square test was used for comparisons.

**Table 5:** Seropositivity rates of both Abs by vaccine type in CRD patients and HCs.

	Ab	CoronaVac	BBIBP-CorV	*p*
CRD patients	Anti-RBD IgG	80.6% (54/67)	55.6% (25/45)	0.004
	CoV-2 NAb	71.6% (48/67)	44.9% (22/45)	0.015
HCs	Anti-RBD IgG	89.5% (51/57)	86.4% (19/22)	0.703
	CoV-2 NAb	84.2% (48/57)	68.2% (15/22)	0.202

The chi-square test was used for comparisons.

**Table 6:** Seropositivity rates for both Abs in CRD patients and HCs (CoronaVac and BBIBP-CorV).

	Ab	CRD patients	HCs	*p*
CoronaVac	Anti-RBD IgG	80.6% (54/67)	89.5% (51/57)	0.171
	CoV-2 NAb	71.6% (48/67)	84.2% (48/57)	0.095
BBIBP-CorV	Anti-RBD IgG	55.6% (25/45)	86.4% (19/22)	0.013
	CoV-2 NAb	44.9% (22/45)	68.2% (15/22)	0.136

The chi-square test was used for comparisons.

**Table 7:** Seropositivity rates of both Abs in CRD subgroups***.***

Ab vaccine	CB Patients	OPTB patients	COPD patients	HCs
Anti-RBD IgG (CoronaVac)	100% (16/16)	69.6% (16/23)*	78.6% (22/28)	89.5% (51/57)
^#^CoV-2 NAb (CoronaVac)	100% (16/16)	60.9% (14/23)*	64.3% (18/28)	84.2% (48/57)
Anti-RBD IgG (BBIBP-CorV)	63.2% (12/19)	54.5% (6/11)	46.7% (7/15)*	86.4% (19/22)
CoV-2 NAb (BBIBP-CorV)	52.6% (10/19)	54.5% (6/11)	40.0% (6/15)	68.2% (15/22)

The chi-square, Fisher's exact, and Dunn's multiple comparisons tests were used for comparisons (*p < 0.05 vs. HCs, ^#^*p* < 0.05 CB vs. OPTB vs. COPD).

**Table 8:** Seropositivity rates of both Abs in CRD patients and HCs aged ≥60 and <60 years.

	Ab (vaccine type)	Age ≥ 60 years	Age < 60 years	*p*
CRD patients	Anti-RBD IgG (BBIBP-CorV)	50.0% (13/26)	63.2% (12/19)	0.380
	Cov-2 NAb (BBIBP-CorV)	42.3% (11/26)	57.9% (11/19)	0.302
	Anti-RBD IgG (CoronaVac)	83.0% (39/47)	75.0% (15/20)	0.676
	CoV-2 NAb (CoronaVac)	72.3% (34/47)	70.0% (14/20)	0.846
HCs	Anti-RBD IgG (CoronaVac)	85.0% (34/40)	100.0% (17/17)	1.000
	CoV-2 NAb (CoronaVac)	80.0% (32/40)	94.1% (16/17)	0.347
	Anti-RBD IgG (BBIBP-CorV)	83.3% (10/12)	90.0% (9/10)	1.000
	CoV-2 NAb (BBIBP-CorV)	75.0% (9/12)	60.0% (6/10)	0.770

The chi-square test was used for comparisons.

**Table 9:** Univariate and multivariate analyses for anti-RBD IgG Ab in CRD.

	Univariate OR (95%CI)	*P*	Multivariate OR (95%CI)	*P*
Gender (female)	1.622(0.711~3.822)	0.257	2.291(0.753~7.399)	0.151
Age (years)	0.998(0.964~1.035)	0.924	1.035(0.985~1.092)	0.185
BMI(Kg/m^2)	1.122(0.982~1.292)	0.096	1.161(0.974~1.408)	0.108
Days after 2^nd^ dose	1.023(1.010~1.037)	0.001	1.030(1.014~1.050)	<0.001
Vaccine type (BBIBP-corV)	0.301(0.127~0.692)	0.005	0.216(0.064~0.672)	0.010
HB (g/L)	1.004(0.976~1.035)	0.772		
WBC (10^^9^/L)	1.326(1.037~1.730)	0.029	1.252(0.915~1.771)	0.178
LC (10^^9^/L)	1.596(0.798~3.276)	0.190		
PLT (10^^9^/L)	1.003 (0.997~1.008)	0.314		
AST (IU/L)	1.001(0.962~1.035)	0.957		
ALT (IU/L)	1.026(0.975~1.084)	0.320		
**Diseases**				
OPTB	0.411(0.126~1.229)	0.121	0.426(0.091~1.862)	0.260
COPD	1.027(0.402~2.654)	0.956	0.495(0.123~1.916)	0.310
RBD^+^ resting MBCs (%)	0.983(0.941~1.023)	0.419	3.885(0.083~19.540)	0.800
RBD^+^ activated MBCs (%)	1.020(0.984~1.056)	0.270	3.854(0.052~19.518)	0.801
RBD^+^ atypical MBCs (%)	1.022(0.995~1.051)	0.116	6.213(0.033~39.413)	0.737
RBD^+^ intermediate MBCs (%)	0.977(0.951~1.003)	0.082	5.957(0.045~37.667)	0.743
RBD-specific memory B cells (MBCs) (%)	1.006(0.977~1.035)	0.694	1.556(0.747~4.893)	0.366

Abbreviations: BMI, body mass index; WBC, white blood cell; HB, hemoglobin; LC, lymphocyte; PLT, platelet; AST, aspartate transaminase, ALT, alanine aminotransferase; RBD, receptor binding domain; MBC, memory B cell; CI, confidential interval; OR, odds ratio.

**Table 10:** Univariate and multivariate analyses for CoV-2 NAb in CRD.

Variable	Univariate OR (95%CI)	*P*	Multivariate OR (95%CI)	*P*
Gender (female)	1.800(0.828~4.014)	0.143	2.968(1.098~8.600)	0.037
Age (years)	1.013(0.980~1.049)	0.455	1.042(0.998~1.092)	0.070
BMI(Kg/m^2)	1.054(0.930~1.198)	0.411	1.056(0.905~1.240)	0.492
Days after 2^nd^ dose	1.011(1.000~1.024)	0.063	1.016(1.002~1.032)	0.032
Vaccine type (BBIBP-corV)	0.379(0.170~0.828)	0.016	0.267(0.092~0.733)	0.012
HB (g/L)	1.006(0.979~1.034)	0.683		
WBC (10^^9^/L)	1.238(0.982~1.585)	0.078	1.105(0.834~1.490)	0.496
LC (10^^9^/L)	1.173(0.610~2.273)	0.630		
PLT (10^^9^/L)	1.002(0.997~1.007)	0.476		
AST (IU/L)	0.994(0.955~1.027)	0.736		
ALT (IU/L)	1.016(0.967~1.070)	0.518		
Diseases				
OPTB	0.346(0.113~0.978)	0.051	0.371(0.095~1.361)	0.141
COPD	1.159(0.472~2.871)	0.747	0.688(0.208~2.228)	0.533
RBD^+^ resting MBCs (%)	0.977(0.937~1.015)	0.244	0.182(0.099~21.374)	0.720
RBD^+^ activated MBCs (%)	1.022(0.988~1.058)	0.211	0.178(0.039~21.431)	0.716
RBD^+^ atypical MBCs (%)	1.029(1.002~1.058)	0.039	0.173(0.076~24.704)	0.716
RBD^+^ intermediate MBCs (%)	0.974(0.949~0.998)	0.038	0.163(0.031~23.010)	0.708
RBD-specific memory B cells (MBCs) (%)	1.002(0.975~1.030)	0.861	0.922(0.534~1.698)	0.778

Abbreviations: BMI, body mass index; WBC, white blood cell; HB, hemoglobin; LC, lymphocyte; PLT, platelet; AST, aspartate transaminase, ALT, alanine aminotransferase; RBD, receptor binding domain; MBC, memory B cell; CI, confidential interval; OR, odds ratio.
