# Supplementary material for: Immune responses to inactivated COVID-19 vaccine were decreased in Chinese patients with chronic respiratory diseases
Source: Int J Med Sci. 2023 Apr 23;20(6):737–48. doi: 10.7150/ijms.78766 (PMC10198143; doi:10.7150/ijms.78766)
Supplement: Supplementary file 1 — Supplementary figures. [file ijmsv20p0737s1.pdf]

Supplementary Figure 1A-E Memory B cells responses to inactivated vaccines in CRD at 1, 2, 3 month.

Supplementary Figure 1 A-E, the frequencies of RBD^+ resting MBCs, RBD^+ activated MBCs, RBD^+ atypical MBCs, RBD^+ intermediate MBCs, and RBD-specific memory B cells (MBCs) were determined. The Mann-Whitney U test was used to compare the frequencies of MBCs.

Supplementary Figure 2A-K. Humoral immune responses to inactivated SARS-CoV-2 vaccines in CRD subgroups.

The titers of anti-RBD IgG Abs and CoV-2 NAbs in the CB, OPTB and COPD patients and HCs after vaccination are shown in **supplementary Figure 2A, B**(Corona Vac) and **C, D**(BBIBP-CorV). **Supplementary Figure 2 E-I**(Corona Vac) shows, the frequencies of RBD^+ resting MBCs, RBD^+ activated MBCs, RBD^+ atypical MBCs, RBD^+ intermediate MBCs and RBD-specific MBCs in the CB, OPTB and COPD patients and HCs. **Supplementary Figure 2 J-K**(BBIBP-CorV) shows, the frequencies of RBD^+ resting MBCs and RBD-specific MBCs in the CB, OPTB and COPD patients and HCs. The Kruskal–Wallis test and Dunn’s multiple comparisons test were employed to compare the Ab titers and the frequencies of MBCs.

Supplementary Figure 3A-J. Humoral immune responses to inactivated vaccines aged ≥60 and <60 years.

The titers of The titers of anti-RBD IgG Abs and CoV-2 NAbs in the CRD patients and HCs aged ≥60 and <60 years after vaccination are shown in **supplementary**

Figure 3A, B(Corona Vac) and **C, D**(BBIBP-CorV). **Supplementary Figure 3E-H**(BBIBP-CorV) shows, the frequencies of RBD$^+$ resting MBCs, RBD$^+$ activated MBCs, RBD$^+$ atypical MBCs and RBD-specific memory B cells(MBCs) in CRD patients and HCs aged ≥60 and <60 years. **Supplementary Figure 3I-J**(Corona Vac) shows, the frequencies of RBD$^+$ resting MBCs and RBD-specific memory B cells(MBCs) in CRD patients and HCs aged ≥60 and <60 years. The T test, Mann-Whitney U test were used to comparethe Ab titers and the frequencies of MBCs.

Supplementary Figure 1A-E

A

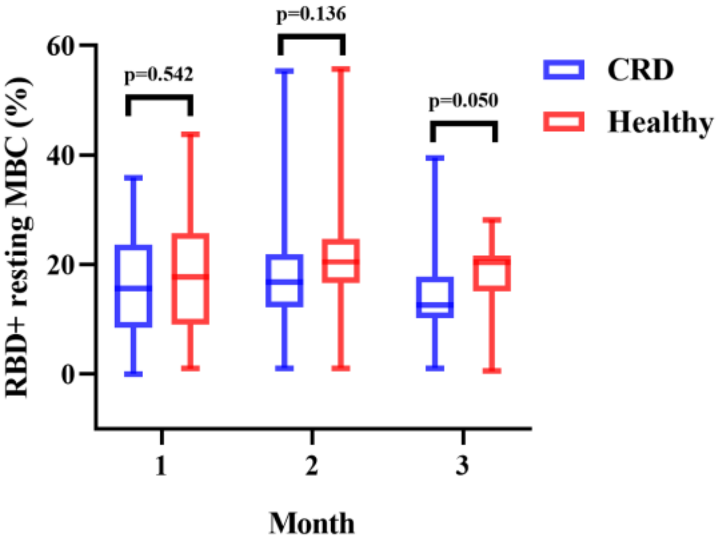

B

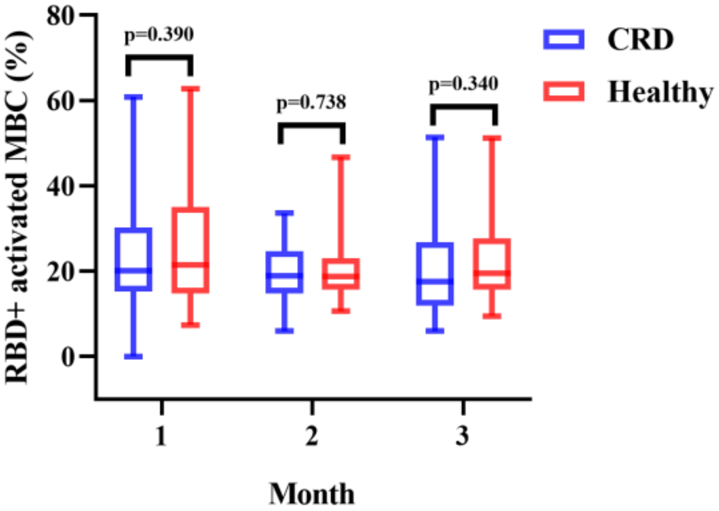

C

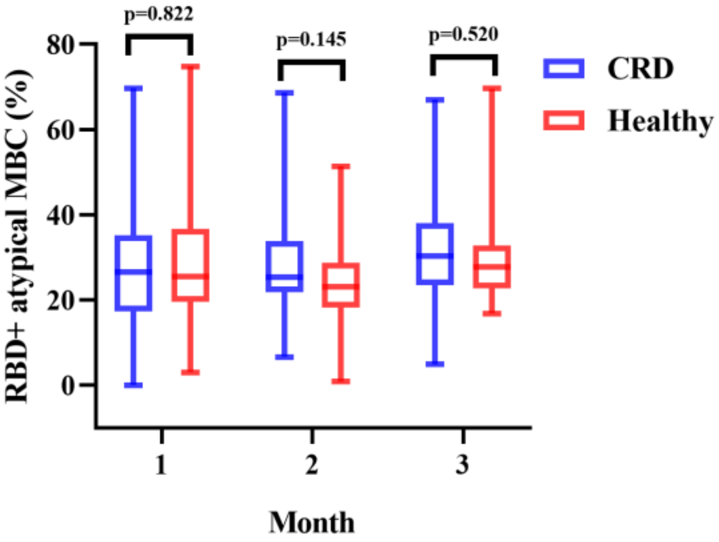
p=0.822
p=0.145
p=0.520
CRD
Healthy
RBD+ atypical MBC (%)
80
60
40
20
0
1
2
3
Month

D

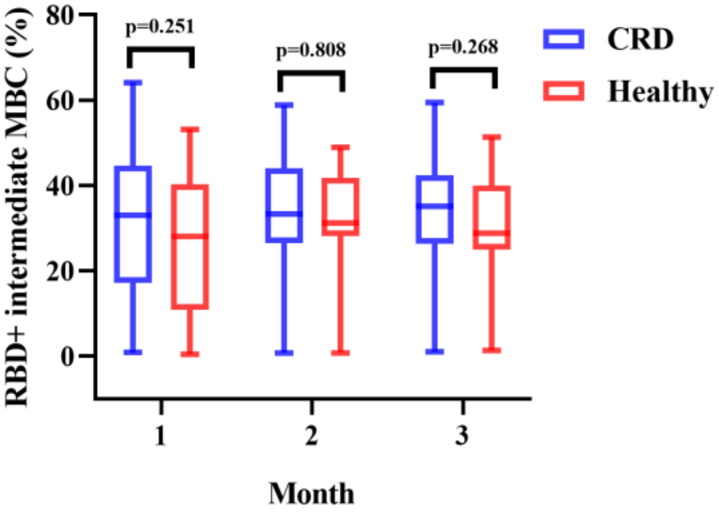
p=0.251
p=0.808
p=0.268
CRD
Healthy
RBD+ intermediate MBC (%)
80
60
40
20
0
1
2
3
Month

E

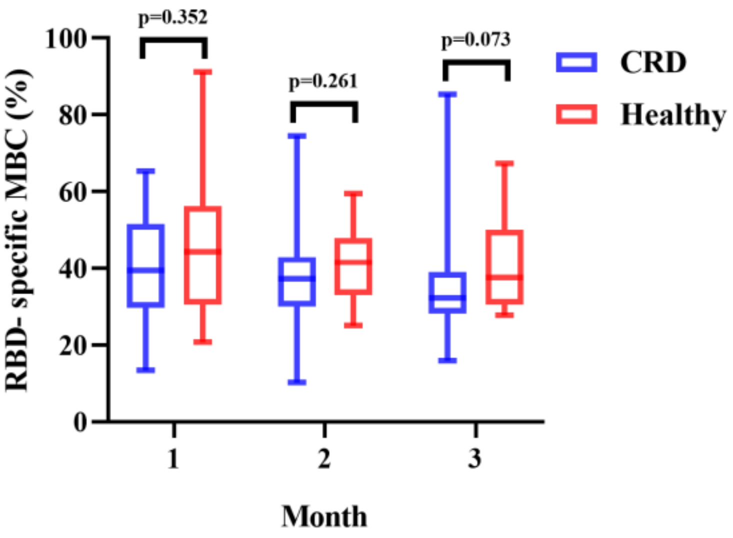

Supplementary Figure 2A-K.

A

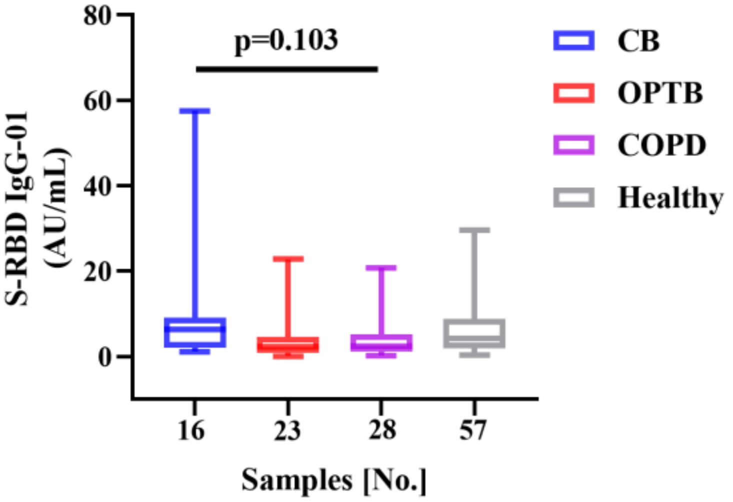

B

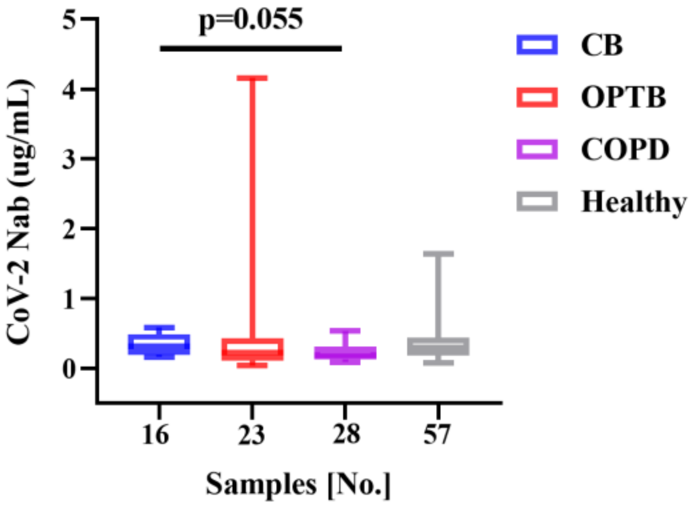

p=0.055
CB
OPTB
COPD
Healthy
CoV-2 Nab (ug/mL)
5
4
3
2
1
0
16
23
28
57
Samples [No.]

C

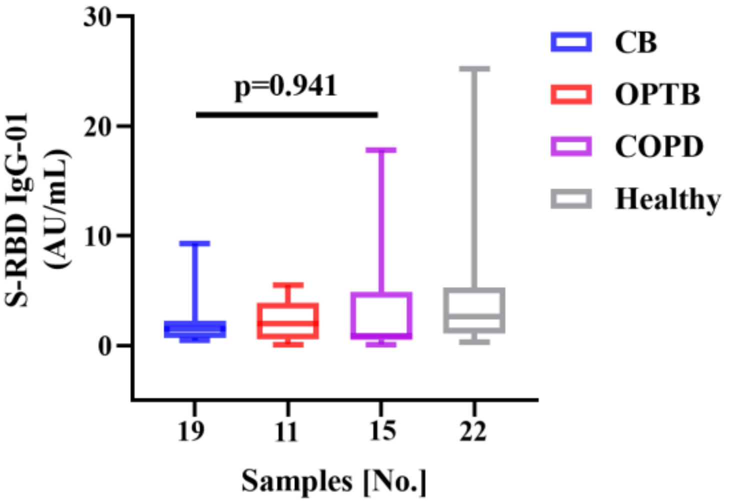

p=0.941
CB
OPTB
COPD
Healthy
S-RBD IgG-01 (AU/mL)
30
20
10
0
19
11
15
22
Samples [No.]

D

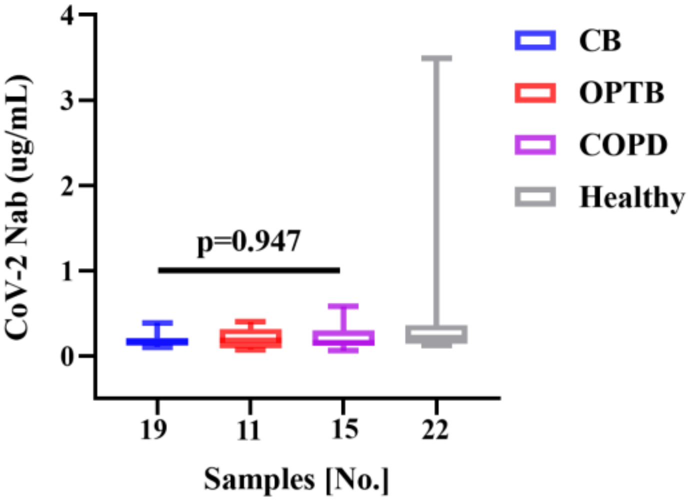

p=0.947
CB
OPTB
COPD
Healthy
CoV-2 Nab (ug/mL)
4
3
2
1
0
19
11
15
22
Samples [No.]

E

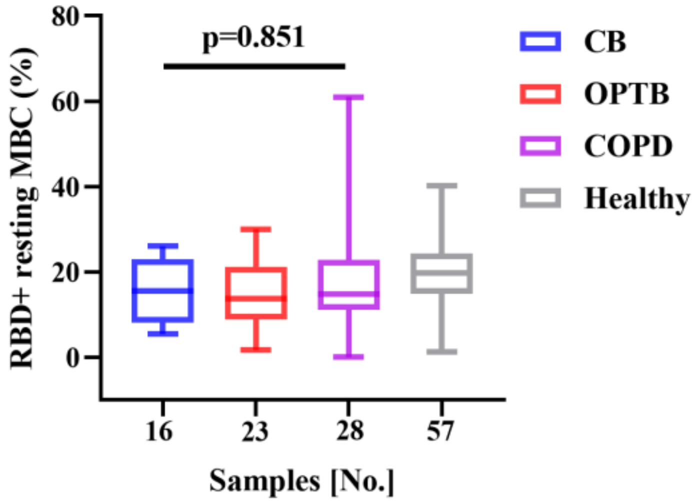

80
60
40
20
0
p=0.851
RBD+ resting MBC (%)
16
23
28
57
Samples [No.]
CB
OPTB
COPD
Healthy

F

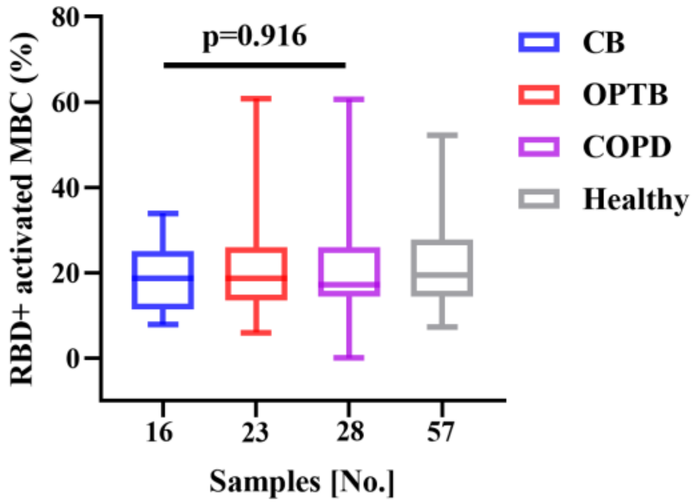

80
60
40
20
0
p=0.916
RBD+ activated MBC (%)
16
23
28
57
Samples [No.]
CB
OPTB
COPD
Healthy

G

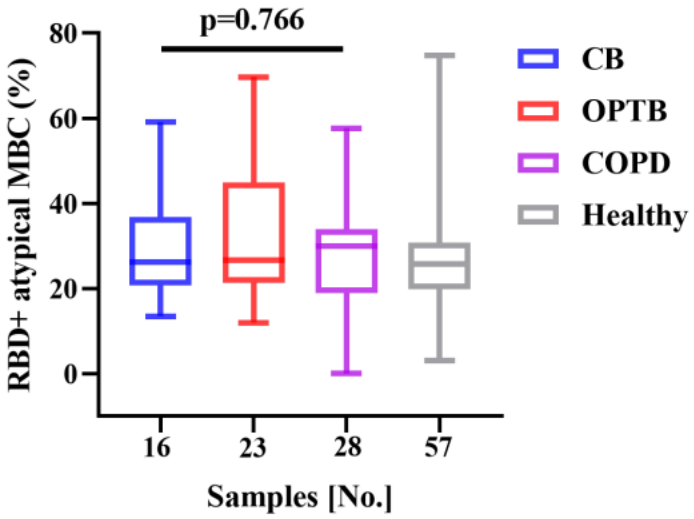
p=0.766
80
60
40
20
0
RBD+ atypical MBC (%)
16
23
28
57
Samples [No.]
CB
OPTB
COPD
Healthy

H

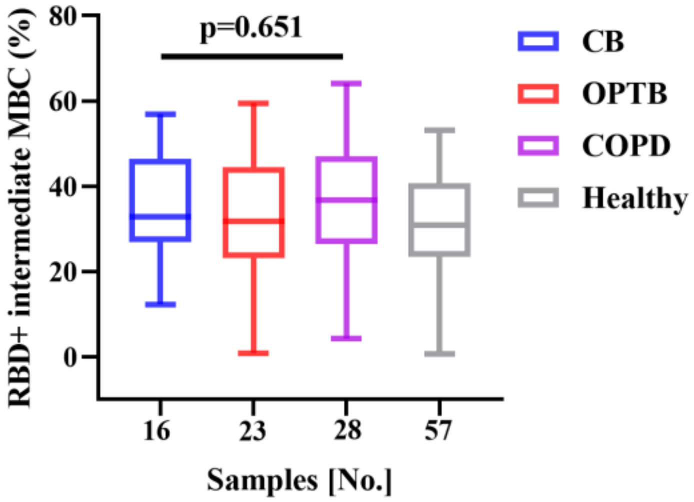
p=0.651
80
60
40
20
0
RBD+ intermediate MBC (%)
16
23
28
57
Samples [No.]
CB
OPTB
COPD
Healthy

I

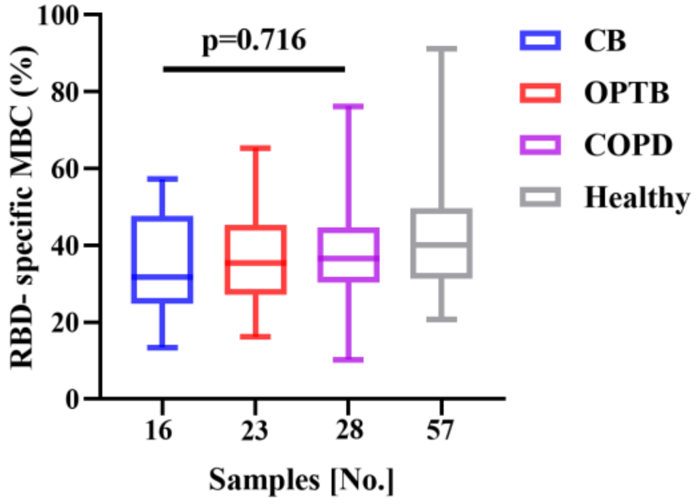
p=0.716
CB
OPTB
COPD
Healthy
RBD- specific MBC (%)
100
80
60
40
20
0
16
23
28
57
Samples [No.]

J

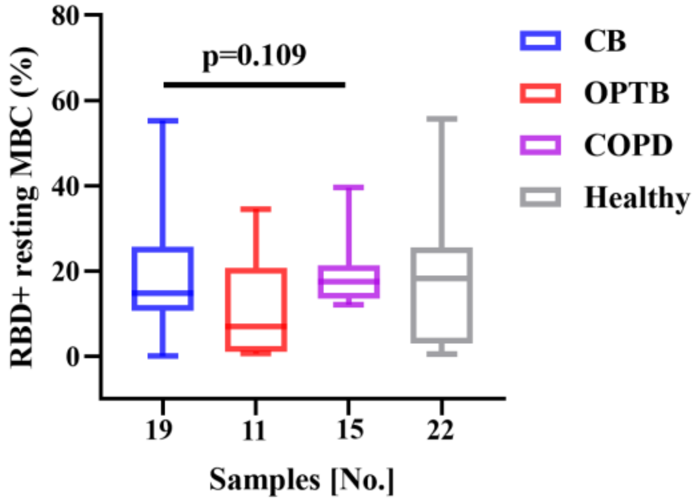
p=0.109
CB
OPTB
COPD
Healthy
RBD+ resting MBC (%)
80
60
40
20
0
19
11
15
22
Samples [No.]

K

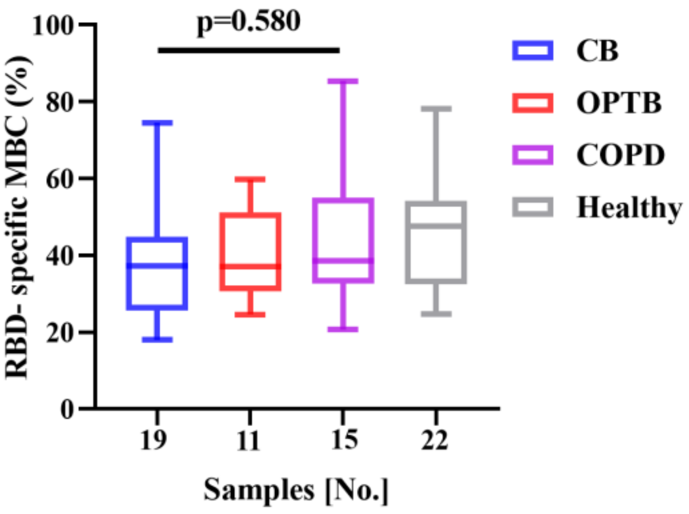

Supplementary Figure 3A-J.

A

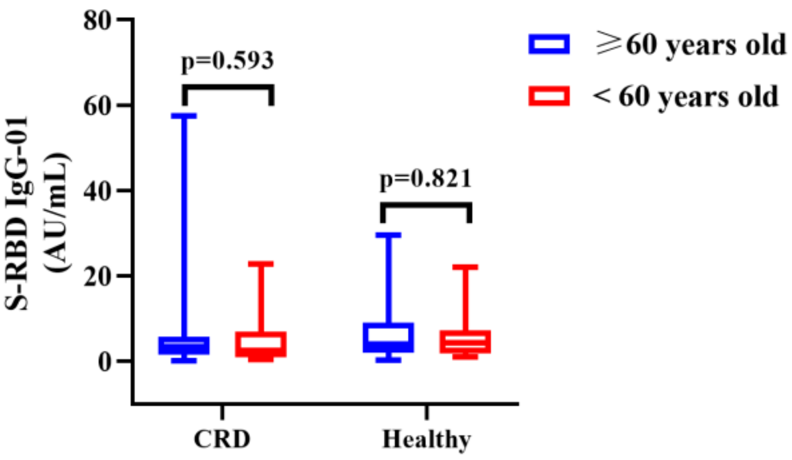

B

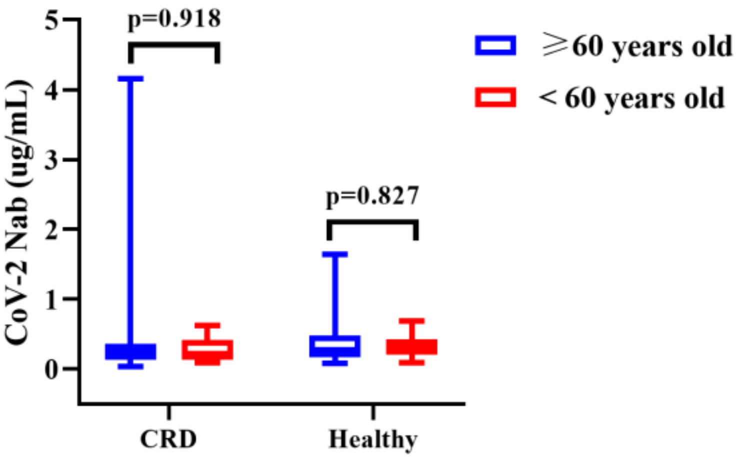
p=0.918
p=0.827
CoV-2 Nab (ug/mL)
0
1
2
3
4
5
CRD
Healthy
⩾60 years old
< 60 years old

C

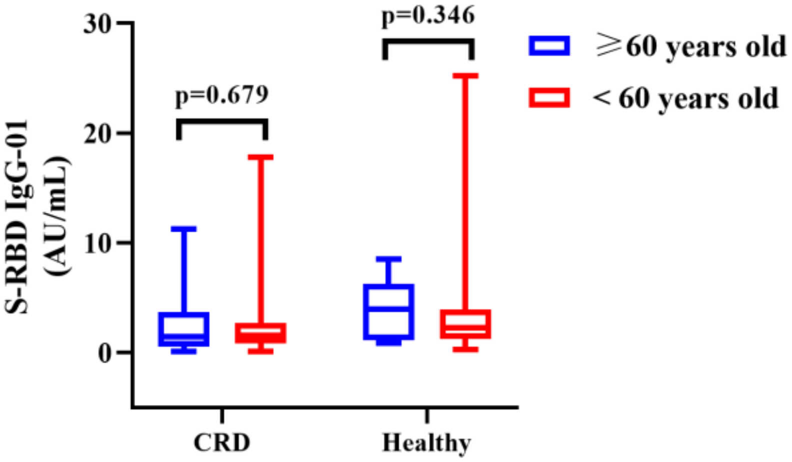
p=0.679
p=0.346
S-RBD IgG-01 (AU/mL)
0
10
20
30
CRD
Healthy
⩾60 years old
< 60 years old

D

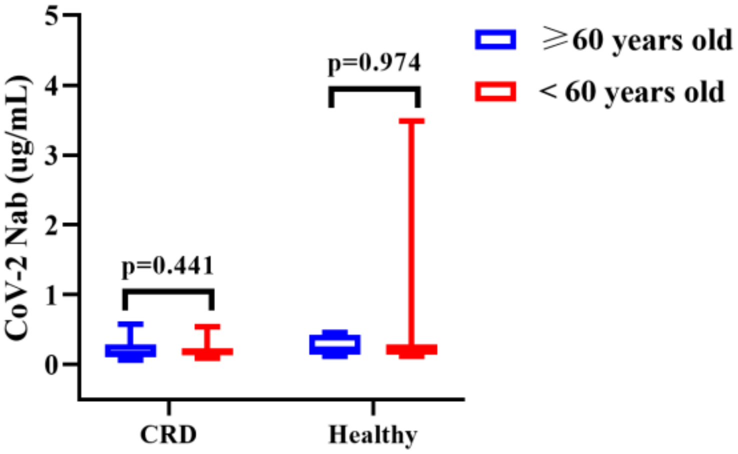
CoV-2 Nab (ug/mL)
5
4
3
2
1
0
p=0.441
p=0.974
CRD
Healthy
⩾60 years old
< 60 years old

E

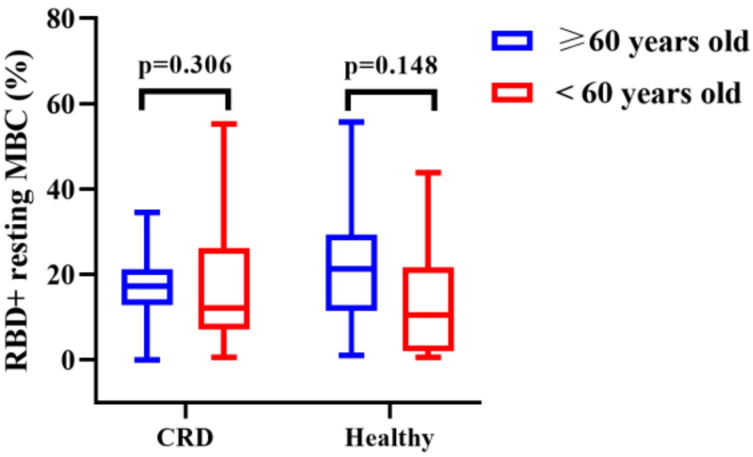
RBD+ resting MBC (%)
80
60
40
20
0
p=0.306
p=0.148
CRD
Healthy
⩾60 years old
< 60 years old

F

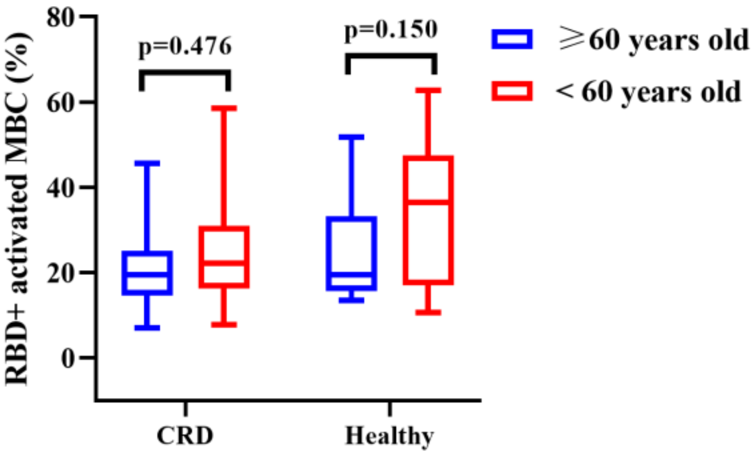
RBD+ activated MBC (%)
80
60
40
20
0
p=0.476
p=0.150
CRD
Healthy
⩾60 years old
< 60 years old

G

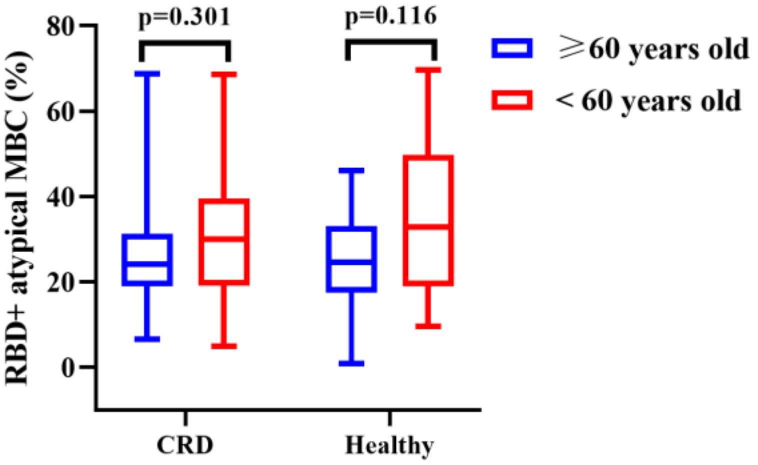
p=0.301
p=0.116
≥60 years old
< 60 years old
RBD+ atypical MBC (%)
80
60
40
20
0
CRD
Healthy

H

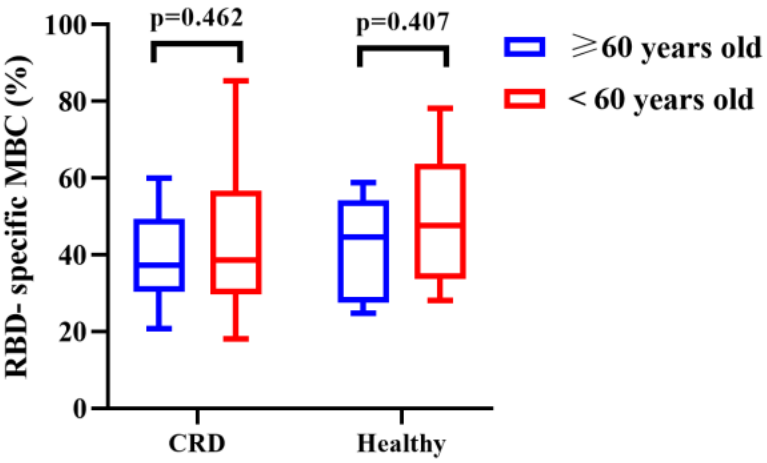
p=0.462
p=0.407
≥60 years old
< 60 years old
RBD- specific MBC (%)
100
80
60
40
20
0
CRD
Healthy

I

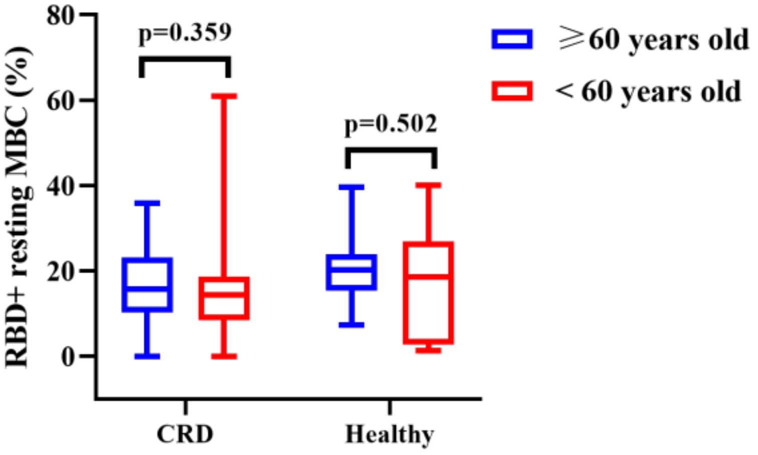
RBD+ resting MBC (%)
80
60
40
20
0
p=0.359
p=0.502
CRD
Healthy
≥60 years old
< 60 years old

J

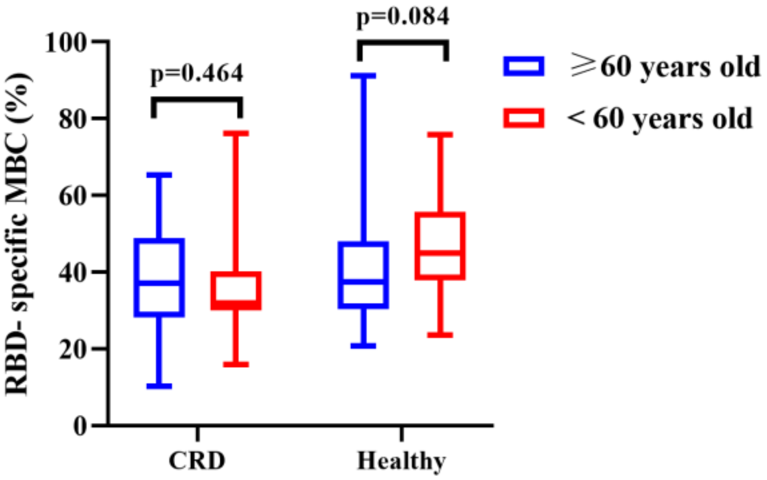
RBD- specific MBC (%)
100
80
60
40
20
0
p=0.464
p=0.084
CRD
Healthy
≥60 years old
< 60 years old
